# Geoeconomic variations in epidemiology, ventilation management, and outcomes in invasively ventilated intensive care unit patients without acute respiratory distress syndrome: a pooled analysis of four observational studies

**DOI:** 10.1016/S2214-109X(21)00485-X

**Published:** 2021-12-13

**Authors:** Luigi Pisani, Anna Geke Algera, Ary Serpa Neto, Luciano Azevedo, Tài Pham, Frederique Paulus, Marcelo Gama de Abreu, Paolo Pelosi, Arjen M Dondorp, Giacomo Bellani, John G Laffey, Marcus J Schultz, ERICC study investigators, Amadeu Martinez, Livia Leal, Antonio Jorge Pereira, Marcelo de Oliveira Maia, Josè Aires Neto, Claudio Piras, Eliana Bernadete Caser, Cora Lavigne Moreira, Pablo Braga Gusman, Dyanne Moysés Dalcomune, Alexandre Guilherme Ribeiro de Carvalho, Louise Aline Romão Gondim, Lívia Mariane Castelo Branco Reis, Daniel da Cunha Ribeiro, Leonardo de Assis Simões, Rafaela Siqueira Campos, José Carlos Fernandez Versiani dos Anjos, Frederico Bruzzi Carvalho, Rossine Ambrosio Alves, Lilian Batista Nunes, Álvaro Réa-Neto, Mirella Cristine de Oliveira, Luana Tannous, Brenno Cardoso Gomes, Fernando Borges Rodriguez, Priscila Abelha, Marcelo E Lugarinho, Andre Japiassu, Hélder Konrad de Melo, Elton Afonso Lopes, Pedro Varaschin, Vicente Cés de Souza Dantas, Marcos Freitas Knibel, Micheli Ponte, Pedro Mendes de Azambuja Rodrigues, Rubens Carmo Costa Filho, Felipe Saddy, Théia Forny Wanderley Castellões, Suzana Alves Silva, Luiz Antonio Gomes Osorio, Dora Mannarino, Rodolfo Espinoza, Cassia Righy, Marcio Soares, Jorge Salluh, Lilian Tanaka, Daniel Aragão, Maria Eduarda Tavares, Maura Goncalves Pereira Kehdi, Valéria Maria Campos Rezende, Roberto Carlos Cruz Carbonell, Cassiano Teixeira, Roselaine Pinheiro de Oliveira, Juçara Gasparetto Maccari, Priscylla Souza Castro, Paula Berto, Patricia Schwarz, André Peretti Torelly, Thiago Lisboa, Edison Moraes, Felipe Dal-Pizzol, Cristiane Tomasi Damiani, Cristiane Ritter, Juliana Carvalho Ferreira, Ramon Teixeira Costa, Pedro Caruso, Cristina Prata Amendola, Amanda Maria R R de Oliveira, Ulysses V A Silva, Luciana Coelho Sanches, Rosana D S Almeida, Luciano Cesar Azevedo, Marcelo Park, Guilherme Schettino, Murillo Santucci Assunção, Eliezer Silva, Carlos Eduardo Barboza, Antonio Paulo Nassar Junior, Paulo Fernando G M Marzocchi Tierno, Luis Marcelo Malbouisson, Lucas Oliveira, Davi Cristovao, Manoel Leitão Neto, Ênio Rego, Fernanda Eugênia Fernandes, Marcelo Luz Pereira Romano, Alexandre Biasi Cavalcanti, Dalton de Souza Barros, Érica Aranha Suzumura, Karla Loureiro Meira, Gustavo Affonso de Oliveira, Paula Menezes Luciano, Evelin Drociunas Pacheco, Bruno Franco Mazza, Flavia Ribeiro Machado, Elaine Ferreira, Ronaldo Batista dos Santos, Alexandra Siqueira Colombo, Antonio Carlos Nogueira, Juliana Baroni Fernandes, Raquel Siqueira Nóbrega, Barbara do CS Martins, Francisco Soriano, Rafaela Deczka Morsch, Andre Luiz Baptiston Nunes, Juliano Pinheiro de Almeida, Ludhmila Hajjar, Sílvia Moulin, Fábio Poianas Giannini, Andre Luiz Baptiston Nunes, LUNG SAFE study investigators, Fernando Rios, Frank Van Haren, T Sottiaux, Fredy S Lora, Luciano C Azevedo, P Depuydt, Eddy Fan, Guillermo Bugedo, Haibo Qiu, Marcos Gonzalez, Juan Silesky, Vladimir Cerny, Jonas Nielsen, Manuel Jibaja, Tài Pham, Hermann Wrigge, Dimitrios Matamis, Jorge Luis Ranero, S M Hashemian, Pravin Amin, Kevin Clarkson, Giacomo Bellani, Kiyoyasu Kurahashi, Asisclo Villagomez, Amine Ali Zeggwagh, Leo M Heunks, Jon Henrik Laake, Jose Emmanuel Palo, Antero do Vale Fernandes, Dorel Sandesc, Yaasen Arabi, Vesna Bumbasierevic, Jose A Lorente, Anders Larsson, Lise Piquilloud, Fekri Abroug, Daniel F McAuley, Lia McNamee, Javier Hurtado, Ed Bajwa, Gabriel Démpaire, Guy M Francois, Hektor Sula, Lordian Nunci, Alma Cani, Alan Zazu, Christian Dellera, Carolina S Insaurralde, Risso V Alejandro, Julio Daldin, Mauricio Vinzio, Ruben O Fernandez, Luis P Cardonnet, Lisandro R Bettini, Mariano Carboni Bisso, Emilio M Osman, Mariano G Setten, Pablo Lovazzano, Javier Alvarez, Veronica Villar, Cesar Milstein, Norberto C Pozo, Nicolas Grubissich, Gustavo A Plotnikow, Daniela N Vasquez, Santiago Ilutovich, Norberto Tiribelli, Ariel Chena, Carlos A Pellegrini, María G Saenz, Elisa Estenssoro, Matias Brizuela, Hernan Gianinetto, Pablo E Gomez, Valeria I Cerrato, Marco G Bezzi, Silvina A Borello, Flavia A Loiacono, Adriana M Fernandez, Serena Knowles, Claire Reynolds, Deborah M Inskip, Jennene J Miller, Jing Kong, Christina Whitehead, Shailesh Bihari, Aylin Seven, Amanda Krstevski, Helen J Rodgers, Rebecca T Millar, Toni E Mckenna, Irene M Bailey, Gabrielle C Hanlon, Anders Aneman, Joan M Lynch, Raman Azad, John Neal, Paul W Woods, Brigit L Roberts, Mark R Kol, Helen S Wong, Katharina C Riss, Thomas Staudinger, Xavier Wittebole, Caroline Berghe, Pierre A Bulpa, Alain M Dive, Rik Verstraete, Herve Lebbinck, Pieter Depuydt, Joris Vermassen, Philippe Meersseman, Helga Ceunen, Jonas I Rosa, Daniel O Beraldo, Claudio Piras, Adenilton M R Ampinelli, Antonio P Nassar Jr, Sergio Mataloun, Marcelo Moock, Marlus M Thompson, Claudio H Gonçalves, Ana Carolina P Antônio, Aline Ascoli, Rodrigo S Biondi, Danielle C Fontenele, Danielle Nobrega, Vanessa M Sales, Suresh Shindhe, Dk Maizatul Aiman B Pg Hj Ismail, John Laffey, Francois Beloncle, Kyle G Davies, Rob Cirone, Venika Manoharan, Mehvish Ismail, Ewan C Goligher, Mandeep Jassal, Erin Nishikawa, Areej Javeed, Gerard Curley, Nuttapol Rittayamai, Matteo Parotto, Niall D Ferguson, Sangeeta Mehta, Jenny Knoll, Antoine Pronovost, Sergio Canestrini, Alejandro R Bruhn, Patricio H Garcia, Felipe A Aliaga, Pamela A Farías, Jacob S Yumha, Claudia A Ortiz, Javier E Salas, Alejandro A Saez, Luis D Vega, Eduardo F Labarca, Felipe T Martinez, Nicolás G Carreño, Pilar Lora, Haitao Liu, Haibo Qiu, Ling Liu, Rui Tang, Xiaoming Luo, Youzhong An, Huiying Zhao, Yan Gao, Zhe Zhai, Zheng L Ye, Wei Wang, Wenwen Li, Qingdong Li, Ruiqiang Zheng, Wenkui Yu, Juanhong Shen, Xinyu Li, Tao Yu, Weihua Lu, Ya Q Wu, Xiao B Huang, Zhenyang He, Yuanhua Lu, Hui Han, Fan Zhang, Renhua Sun, Hua X Wang, Shu H Qin, Bao H Zhu, Jun Zhao, Jian Liu, Bin Li, Jing L Liu, Fa C Zhou, Qiong J Li, Xing Y Zhang, Zhou Li-Xin, Qiang Xin-Hua, Liangyan Jiang, Yuan N Gao, Xian Y Zhao, Yuan Y Li, Xiao L Li, Chunting Wang, Qingchun Yao, Rongguo Yu, Kai Chen, Huanzhang Shao, Bingyu Qin, Qing Q Huang, Wei H Zhu, Ai Y Hang, Ma X Hua, Yimin Li, Yonghao Xu, Yu D Di, Long L Ling, Tie H Qin, Shou H Wang, Junping Qin, Yi Han, Suming Zhou, Monica P Vargas, Juan I Silesky Jimenez, Manuel A González Rojas, Jaime E Solis-Quesada, Christian M Ramirez-Alfaro, Jan Máca, Peter Sklienka, Jakob Gjedsted, Aage Christiansen, Jonas Nielsen, Boris G Villamagua, Miguel Llano, Philippe Burtin, Gautier Buzancais, Pascal Beuret, Nicolas Pelletier, Satar Mortaza, Alain Mercat, Jonathan Chelly, Sébastien Jochmans, Nicolas Terzi, Cédric Daubin, Guillaume Carteaux, Nicolas de Prost, Jean-Daniel Chiche, Fabrice Daviaud, Tai Pham, Muriel Fartoukh, Guillaume Barberet, Jerome Biehler, Jean Dellamonica, Denis Doyen, Jean-Michel Arnal, Anais Briquet, Sami Hraiech, Laurent Papazian, Arnaud Follin, Damien Roux, Jonathan Messika, Evangelos Kalaitzis, Laurence Dangers, Alain Combes, Siu-Ming Au, Gaetan Béduneau, Dorothée Carpentier, Elie H Zogheib, Herve Dupont, Sylvie Ricome, Francesco L Santoli, Sebastien L Besset, Philippe Michel, Bruno Gelée, Pierre-Eric Danin, Bernard Goubaux, Philippe J Crova, Nga T Phan, Frantz Berkelmans, Julio C Badie, Romain Tapponnier, Josette Gally, Samy Khebbeb, Jean-Etienne Herbrecht, Francis Schneider, Pierre-Louis M Declercq, Jean-Philippe Rigaud, Jacques Duranteau, Anatole Harrois, Russell Chabanne, Julien Marin, Charlene Bigot, Sandrine Thibault, Mohammed Ghazi, Messabi Boukhazna, Salem Ould Zein, Jack R Richecoeur, Daniele M Combaux, Fabien Grelon, Charlene Le Moal, Elise P Sauvadet, Adrien Robine, Virginie Lemiale, Danielle Reuter, Martin Dres, Alexandre Demoule, Dany Goldgran-Toledano, Loredana Baboi, Claude Guérin, Ralph Lohner, Jens Kraßler, Susanne Schäfer, Kai D Zacharowski, Patrick Meybohm, Andreas W Reske, Philipp Simon, Hans-Bernd F Hopf, Michael Schuetz, Thomas Baltus, Metaxia N Papanikolaou, Theonymfi G Papavasilopoulou, Giannis A Zacharas, Vasilis Ourailogloy, Eleni K Mouloudi, Eleni V Massa, Eva O Nagy, Electra E Stamou, Ellada V Kiourtzieva, Marina A Oikonomou, Luis E Avila, Cesar A Cortez, Johanna E Citalán, Sameer A Jog, Safal D Sable, Bhagyesh Shah, Mohan Gurjar, Arvind K Baronia, Mohammedfaruk Memon, Radhakrishnan Muthuchellappan, Venkatapura J Ramesh, Anitha Shenoy, Ramesh Unnikrishnan, Subhal B Dixit, Rachana V Rhayakar, Nagarajan Ramakrishnan, Vallish K Bhardwaj, Heera L Mahto, Sudha V Sagar, Vijayanand Palaniswamy, Deeban Ganesan, Seyed Mohammadreza Hashemian, Hamidreza Jamaati, Farshad Heidari, Edel A Meaney, Alistair Nichol, Karl M Knapman, Donall O'Croinin, Eimhin S Dunne, Dorothy M Breen, Kevin P Clarkson, Rola F Jaafar, Rory Dwyer, Fahd Amir, Olaitan O Ajetunmobi, Aogan C O'Muircheartaigh, Colin S Black, Nuala Treanor, Daniel V Collins, Wahid Altaf, Gianluca Zani, Maurizio Fusari, Savino Spadaro, Carlo A Volta, Romano Graziani, Barbara Brunettini, Salvatore Palmese, Paolo Formenti, Michele Umbrello, Andrea Lombardo, Elisabetta Pecci, Marco Botteri, Monica Savioli, Alessandro Protti, Alessia Mattei, Lorenzo Schiavoni, Andrea Tinnirello, Manuel Todeschini, Antonino Giarratano, Andrea Cortegiani, Sara Sher, Anna Rossi, Massimo M Antonelli, Luca M Montini, Paolo Casalena, Sergio Scafetti, Giovanna Panarello, Giovanna Occhipinti, Nicolò Patroniti, Matteo Pozzi, Roberto R Biscione, Michela M Poli, Ferdinando Raimondi, Daniela Albiero, Giulia Crapelli, Eduardo Beck, Vincenzo Pota, Vincenzo Schiavone, Alexandre Molin, Fabio Tarantino, Giacomo Monti, Elena Frati, Lucia Mirabella, Gilda Cinnella, Tommaso Fossali, Riccardo Colombo, Pierpaolo Terragni, Ilaria Pattarino, Francesco Mojoli, Antonio Braschi, Erika E Borotto, Andrea N Cracchiolo, Daniela M Palma, Francesco Raponi, Giuseppe Foti, Ettore R Vascotto, Andrea Coppadoro, Luca Brazzi, Leda Floris, Giorgio A Iotti, Aaron Venti, Osamu Yamaguchi, Shunsuke Takagi, Hiroki N Maeyama, Eizo Watanabe, Yoshihiro Yamaji, Kazuyoshi Shimizu, Kyoko Shiozaki, Satoru Futami, Sekine Ryosuke, Koji Saito, Yoshinobu Kameyama, Keiko Ueno, Masayo Izawa, Nao Okuda, Hiroyuki Suzuki, Tomofumi Harasawa, Michitaka Nasu, Tadaaki Takada, Fumihito Ito, Shin Nunomiya, Kansuke Koyama, Toshikazu Abe, Kohkichi Andoh, Kohei Kusumoto, Akira Hirata, Akihiro Takaba, Hiroyasu Kimura, Shuhei Matsumoto, Ushio Higashijima, Hiroyuki Honda, Nobumasa Aoki, Hiroshi Imai, Yasuaki Ogino, Ichiko Mizuguchi, Kazuya Ichikado, Kenichi Nitta, Katsunori Mochizuki, Tomoaki Hashida, Hiroyuki Tanaka, Tomoyuki Nakamura, Daisuke Niimi, Takeshi Ueda, Yozo Kashiwa, Akinori Uchiyama, Olegs Sabelnikovs, Peteris Oss, Youssef Haddad, Kong Y Liew, Silvio A Ñamendys-Silva, Yves D Jarquin-Badiola, Luis A Sanchez-Hurtado, Saira S Gomez-Flores, Maria C Marin, Asisclo J Villagomez, Jordana S Lemus, Jonathan M Fierro, Mavy Ramirez Cervantes, Francisco Javier Flores Mejia, Daniel R Gonzalez, Dulce M Dector, Claudia R Estrella, Jorge R Sanchez-Medina, Alvaro Ramirez-Gutierrez, Fernando G George, Janet S Aguirre, Juan A Buensuseso, Manuel Poblano, Tarek Dendane, Amine Ali Zeggwagh, Hicham Balkhi, Mina Elkhayari, Nacer Samkaoui, Hanane Ezzouine, Abdellatif Benslama, Mourad Amor, Wajdi Maazouzi, Nedim Cimic, Oliver Beck, Monique M Bruns, Jeroen A Schouten, Myra Rinia, Monique Raaijmakers, Leo M Heunks, Hellen M Van Wezel, Serge J Heines, Marc P Buise, Fabienne D Simonis, Marcus J Schultz, Jennifer C Goodson, Troy S B rowne, Leanlove Navarra, Anna Hunt, Robyn A Hutchison, Mathew B Bailey, Lynette Newby, Colin Mcarthur, Michael Kalkoff, Alex Mcleod, Jonathan Casement, Danielle J Hacking, Finn H Andersen, Merete S Dolva, Jon H Laake, Andreas Barratt-Due, Kim Andre L Noremark, Eldar Søreide, Brit Å Sjøbø, Anne B Guttormsen, Hector H Leon Yoshido, Ronald Zumaran Aguilar, Fredy A Montes Oscanoa, Alain U Alisasis, Joanne B Robles, Rossini Abbie B Pasanting-Lim, Beatriz C Tan, Pawel Andruszkiewicz, Karina Jakubowska, Cristina M Cox, António M Alvarez, Bruno S Oliveira, Gustavo M Montanha, Nelson C Barros, Carlos S Pereira, António M Messias, Jorge M Monteiro, Ana M Araujo, Nuno T Catorze, Susan M Marum, Maria J Bouw, Rui M Gomes, Vania A Brito, Silvia Castro, Joana M Estilita, Filipa M Barros, Isabel M Serra, Aurelia M Martinho, Dana R Tomescu, Alexandra Marcu, Ovidiu H Bedreag, Marius Papurica, Dan E Corneci, Silvius Ioan Negoita, Evgeny Grigoriev, Alexey I Gritsan, Andrey A Gazenkampf, Ghaleb Almekhlafi, Mohamad M Albarrak, Ghanem M Mustafa, Khalid A Maghrabi, Nawal Salahuddin, Tharwat M Aisa, Ahmed S Al Jabbary, Edgardo Tabhan, Yaseen M Arabi, Olivia A Trinidad, Hasan M Al Dorzi, Edgardo E Tabhan, Stefan Bolon, Oliver Smith, Jordi Mancebo, Hernan Aguirre-Bermeo, Juan C Lopez-Delgado, Francisco Esteve, Gemma Rialp, Catalina Forteza, Candelaria De Haro, Antonio Artigas, Guillermo M Albaiceta, Sara De Cima-Iglesias, Leticia Seoane-Quiroga, Alexandra Ceniceros-Barros, Antonio L Ruiz-Aguilar, Luis M Claraco-Vega, Juan Alfonso Soler, Maria del Carmen Lorente, Cecilia Hermosa, Federico Gordo, Miryam Prieto-González, Juan B López-Messa, Manuel P Perez, Cesar P Pere, Raquel Montoiro Allue, Ferran Roche-Campo, Marcos Ibañez-Santacruz, Susana Temprano, Maria C Pintado, Raul De Pablo, Pilar Ricart Aroa Gómez, Silvia Rodriguez Ruiz, Silvia Iglesias Moles, Mª Teresa Jurado, Alfons Arizmendi, Enrique A Piacentini, Nieves Franco, Teresa Honrubia, Meisy Perez Cheng, Elena Perez Losada, Javier Blanco, Luis J Yuste, Cecilia Carbayo-Gorriz, Francisca G Cazorla-Barranquero, Javier G Alonso, Rosa S Alda, Ángela Algaba, Gonzalo Navarro, Enrique Cereijo, Esther Diaz-Rodriguez, Diego Pastor Marcos, Laura Alvarez Montero, Luis Herrera Para, Roberto Jimenez Sanchez, Miguel Angel Blasco Navalpotro, Ricardo Diaz Abad, Raquel Montiel González, Dácil Parrilla Toribio, Alejandro G Castro, Maria Jose D Artiga, Oscar Penuelas, Tomas P Roser, Moreno F Olga, Elena Gallego Curto, Rocío Manzano Sánchez, Vallverdu P Imma, Garcia M Elisabet, Laura Claverias, Monica Magret, Ana M Pellicer, Lucia L Rodriguez, Jesús Sánchez-Ballesteros, Ángela González-Salamanca, Antonio G Jimenez, Francisco P Huerta, Juan Carlos J Sotillo Diaz, Esther Bermejo Lopez, David D Llinares Moya, Alec A Tallet Alfonso, Palazon Sanchez Eugenio Luis, Palazon Sanchez Cesar, Sánchez I Rafael, Corcoles G Virgilio, Noelia N Recio, Richard O Adamsson, Christian C Rylander, Bernhard Holzgraefe, Lars M Broman, Joanna Wessbergh, Linnea Persson, Fredrik Schiöler, Hans Kedelv, Anna Oscarsson Tibblin, Henrik Appelberg, Lars Hedlund, Johan Helleberg, Karin E Eriksson, Rita Glietsch, Niklas Larsson, Ingela Nygren, Silvia L Nunes, Anna-Karin Morin, Thomas Kander, Anne Adolfsson, Lise Piquilloud, Hervé O Zender, Corinne Leemann-Refondini, Souheil Elatrous, Slaheddine Bouchoucha, Imed Chouchene, Islem Ouanes, Asma Ben Souissi, Salma Kamoun, Oktay Demirkiran, Mustafa Aker, Emre Erbabacan, Ilkay Ceylan, Nermin Kelebek Girgin, Menekse Ozcelik, Necmettin Ünal, Basak Ceyda Meco, Onat O Akyol, Suleyman S Derman, Barry Kennedy, Ken Parhar, Latha Srinivasa, Lia McNamee, Danny McAuley, Jack Steinberg, Phil Hopkins, Clare Mellis, Frank Stansil, Vivek Kakar, Dan Hadfield, Christine Brown, Andre Vercueil, Kaushik Bhowmick, Sally K Humphreys, Andrew Ferguson, Raymond Mckee, Ashok S Raj, Danielle A Fawkes, Philip Watt, Linda Twohey, Rajeev R Jha Matthew Thomas, Alex Morton, Varsha Kadaba, Mark J Smith, Anil P Hormis, Santhana G Kannan, Miriam Namih, Henrik Reschreiter, Julie Camsooksai, Alek Kumar, Szabolcs Rugonfalvi, Christopher Nutt, Orla Oneill, Colette Seasman, Ged Dempsey, Christopher J Scott, Helen E Ellis, Stuart Mckechnie, Paula J Hutton, Nora N Di Tomasso, Michela N Vitale, Ruth O Griffin, Michael N Dean, Julius H Cranshaw, Emma L Willett, Nicholas Ioannou, Sarah Gillis, Peter Csabi, Rosaleen Macfadyen, Heidi Dawson, Pieter D Preez, Alexandra J Williams, Owen Boyd, Laura Ortiz-Ruiz De Gordoa, Jon Bramall, Sophie Symmonds, Simon K Chau, Tim Wenham, Tamas Szakmany, Piroska Toth-Tarsoly, Katie H Mccalman, Peter Alexander, Lorraine Stephenson, Thomas Collyer, Rhiannon Chapman, Raphael Cooper, Russell M Allan, Malcolm Sim, David W Wrathall, Donald A Irvine, Kim S Zantua, John C Adams, Andrew J Burtenshaw, Gareth P Sellors, Ingeborg D Welters, Karen E Williams, Robert J Hessell, Matthew G Oldroyd, Ceri E Battle, Suresh Pillai, Istvan Kajtor, Mageswaran Sivashanmugave, Sinead C Okane, Adrian Donnelly, Aniko D Frigyik, Jon P Careless, Martin M May, Richard Stewart, T John Trinder, Samantha J Hagan, Matt P Wise, Jade M Cole, Caroline C MacFie, Anna T Dowling, Javier Hurtado, Nicolás Nin, Javier Hurtado, Edgardo Nuñez, Gustavo Pittini, Ruben Rodriguez, María C Imperio, Cristina Santos, Ana G França, Alejandro Ebeid, Alberto Deicas, Carolina Serra, Aditya Uppalapati, Ghassan Kamel, Valerie M Banner-Goodspeed, Jeremy R Beitler, Satyanarayana Reddy Mukkera, Shreedhar Kulkarni, Jarone Lee, Tomaz Mesar, John O Shinn Iii, Dina Gomaa, Christopher Tainter, Tomaz Mesar, R Adams Cowley, Dale J Yeatts, Jessica Warren, Michael J Lanspa, Russel R Miller, Colin K Grissom, Samuel M Brown, Philippe R Bauer, Ryan J Gosselin, Barrett T Kitch, Jason E Cohen, Scott H Beegle, Renaud M Gueret, Aiman Tulaimat, Shazia Choudry, William Stigler, Hitesh Batra, Nidhi G Huff, Keith D Lamb, Trevor W Oetting, Nicholas M Mohr, Claine Judy, Shigeki Saito, Fayez M Kheir, Adam B Schlichting, Angela Delsing, Mary Elmasri, Daniel R Crouch, Dina Ismail, Thomas C Blakeman, Kyle R Dreyer, Dina Gomaa, Rebecca M Baron, Carolina Quintana Grijalba, Peter C Hou, Raghu Seethala, Imo Aisiku, Galen Henderson, Gyorgy Frendl, Sen-Kuang Hou, Robert L Owens, Ashley Schomer, Vesna Bumbasirevic, Bojan Jovanovic, Maja Surbatovic, Milic Veljovic, PRoVENT study investigators, Frank Van Haren, Helen Rodgers, Barry Dixon, Roger Smith, Mark Kol, Helen Wong, Werner Schmid, Greet Hermans, Helga Ceunen, Marc Bourgeois, Nathalie Anquez, Johan Decruyenaere, Luc DeCrop, Ary Serpa Neto, Rafaella Souza dos Santos, Daniel Beraldo, Moreno Calcagnotto dos Santos, Jose Augusto Santos Pellegrini, Claudio Piras, Vanessa Oliveira, Carlos Munhoz, Ana Carolina Peçanha, Fernando José da Silva Ramos, Israel Maia, Marina Bahl, Rodrigo Biondi, Daniel Prado, Sérgio Felix Pinto, Jean Salgado, Luis Fernando Falcão, Tiago Macruz, Alexandre Biasi Cavalcanti, Marcelo Luz Pereira Romano, Kessia Ruas, Giovana Colozza Mecatti, Eliane Bernadete Caser, Isabela Ambrósio Gava, Nicolás Carreño, Mauricio Morales, Rossana Avendaño, Stefania Aguirre, Andrej Sribar, Vlasta Klaric, Sonja Skilijic, Matea Bogdanovic Dvorscak, Marijana Krkusek, Matija Jurjevic, Nenad Karanovic, Tatjana Simurina, Petr Stourac, Milan Kratochvil, Jan Máca, Hermann Wrigge, Christian Schlegel, Tanja A Treschan, Maximilian Schaefer, Akut Aytulun, Peter Kienbaum, Kevin Clarkson, Rola Jaafar, Daniel Collins, Robert Plant, Giuseppe Melchionda, Eduardo Di Lauro, Andrea Cortegiani, Vincenzo Russotto, Raffaele Caione, Donatella Mestria, Carlo Alberto Volta, Savino Spadaro, Marco Botteri, Elisa Seghelini, Luca Brazzi, Gabriele Sales, Davide D'Antini, Alexandre Molin, Paolo Severgnini, Alessandro Bacuzzi, Lorenzo Peluso, Pasquale Verrastro, Pasquale Raimondo, Agreta Gecaj-Gashi, Fabienne D Simonis, Pieter Roel Tuinman, Erna Alberts, Ingrid van den Hul, Michael Kuiper, Robert BP de Wilde, Matty Koopmans, Isil Kose, Çiler Zincircioglu, Nazim Dogan, Demet Aydin, Ahmet Sukru Denker, Unase Buyukkocak, Nur Akgun, Güldem Turan, Evren Senturk, Zerrin Demirtürk, Perihan Ergin Özcan, Osman Ekinci, Sedat Saylan, Gulay Eren, Fatma Ulger, Ahmet Dilek, Hulya Ulusoy, Ugur Goktas, Lokman Soyoral, Huseyin Toman, Yavuz Orak, Feda Kahveci, Gary H Mills, Angela Pinder, Rachel Walker, Jonathan Harrison, Jane Snell, Colette Seasman, Rachel Pearson, Michael Sharman, Claire Kaloo, Natalie Bynorth, Kelly Matthews, Chloe Hughes, Alastair Rose, Karen Simeson, Lotta Niska, Nathan Huneke, Jane Adderly, Cheryl Padilla-Harris, Rebecca Oliver, Farooq Brohi, Natalie Wilson, Helen Talbot, Deborah Wilson, Deborah Smith, Paulo Dark, Tracey Evans, Nicola Fisher, Jane Montgomery, Pauline Fitzell, Christoph Muench, Keith Hugill, Emanuel Cirstea, Andrew Bentley, Katie Lynch, Ian White, Jonathan Cooper, Melinda Brazier, Michael Devile, Michael Parris, Pardeep Gill, Tasmin Patel, John Criswell, Dawn Trodd, Denise Griffin, Jane Martin, Caroline Wreybrown, Jeremy Bewley, Katie Sweet, Lisa Grimmer, Marta Kozlowski, Shanaz James, James Limb, Amanda Cowton, David Rogerson, Charlotte Downes, Susan Melbourne, Ryan Humphries, Mark Pulletz, Sarah Moreton, Stephanie Janes, Andrew Corner, Vanessa Linnett, Jenny Ritzema, Malcolm Watters, Steve Windebank, Shailaja Chenna, Richard Howard-Griffin, Kate Turner, Sheeba Suresh, Heather Blaylock, Stephanie Bell, Karl Blenk, Lynn Everett, Phil Hopkins, Clare Mellis, Daniel Hadfield, Clair Harris, Alexandre Chan, Sian Birch, Claire Pegg, Catherine Plowright, Lucy Cooper, Tom Hatton, Iain McCullagh, Stephen Wright, Carmen Scott, Christine Boyd, Mark Holliday, Una Poultney, Hannah Crowther, Sarah Thornthwaite, Nigel Hollister, Jane Hunt, Amanda Skinner, Ramprasad Matsa, Ruth Salt, Claire Matthews, Henrik Reschreiter, Julie Camsooksai, Nicola Venner, Helena Barcraft-Barnes, Lee Tbaily, David Pogson, Johanna Mouland, Steve Rose, Nicola Lamb, Nicholas Tarmey, John Knighton, Julian Giles, Debbie Weller, Isabelle Reed, Anil Hormis, Sallyane Pearson, Meredith Harris, Joanne Howe, Jonathan Paddle, Karen Burt, Ingeborg Welters, Anna Walker, Laura Youds, Sam Hendry, David Shaw, Karen Williams, Robin Hollands, Mandy Carnahan, Johanna Stickley, Claire Miller, Denise Donaldson, Louise Tonks, Ben Creagh-Brown, Daniel Hull, Owen Boyd, Laura Ortiz-Ruiz, Shammer Gopal, Stella Metherell, Hazel Spencer, Christian Frey, Carly Brown, Gayle Clifford, Susannah Leaver, Christine Ryan, Johannes Mellinghoff Mellinghoff, Sarah Prudden Prudden, Helen Green Green, Alistair Roy Roy, Julie Furneval Furneval, Adam Bell Bell, Sandeep Lakhani Lakhani, Lousie Fasting Fasting, Lorna Murray Murray, Kobus Preller, Amy McInerney, Sarah Beavis, Amanda Whileman, Julie Toms, Sue Glenn, Mohamed Ramali, Alison Ghosh, Clare Bullock, Lisa Barrell, Eoin Young, Helen Robertson, Maria Faulkner, Peter MacNaughton, Susan Tyson, Paul Pulak, Terri-Ann Sewell, Christopher Smalley, Reni Jacob, Cristina Santos, Pedro Alzugaray, Marcos F Vidal Melo, Kristen Joyce, Joseph Needleman, PRoVENT-iMiC study investigators, Areef Ahsan, Abul Faiz, AKM Shamsul Alam, Syeda Nafisa Khatoon, Ranjan Kumer Nath, Mohammed Abdur Rahman Chowdhury, Debabrata Banik, Montosh Kumar Mondol, Sakibur Rahman Bhuiyan, Suraiya Nazneed, Rozina Sultana, Tarikul Hamid, Mozaffer Hossain, Syed Tariq Reza, Muhammad Asaduzzaman, Mohammad Salim, Abu Hena Mostafa Kamal, Sheikh Mohammed Taher, Taohidul Majid Taohid, Pranab Karmaker, Sabyasachi Roy, Shantanu Das, Sohel Ahmed Sarkar, Monju Lal Dutta, Poulomi Roy, Shivakumar Iyer, Bhuvana Krishna, Sriram Sampath, Rajyabardhan Pattnaik, Chinni Krishna Kasi, Jignesh Shah, Anand Dongre, Seyed Mohammad Reza Hashemian, Navid Nooraei, Reza Raessi Estabragh, Majid Malekmohammad, Batoul Khoundabi, Maziar Mobasher, Nor'azim Mohd Yunos, Mahazir Kassim, Chern Min Voon, Stanis Sutharsa Das, Siti Nur Suhaila Azauddin, Dharshinie Dorasamy, Li Ling Tai, Mohd Basri Mat Nor, Nurhafizah Zarudin, Mohd Shahnaz Hasan, Mohamad Fadhil Hadi Jamaluddin, Mohamad Irfan Othman Jailani, Gyan Kayashta, Aaradhana Adhikari, Raju Pangeni, Madiha Hashmi, Sonia Joseph, Aftab Akhtar, Aayesha Qadeer, Iqbal Memon, Syed Muneeb Ali, Farah Idrees, Saima Kamal, Sadaf Hanif, Atta Ur Rehman, Arshad Taqi, Tanveer Hussain, Ahmed Farooq, Saleh Khaskheli, Muhammad Hayat, Kanishka Indraratna, Abigail Beane, Rashan Haniffa, Upeka Samaranayake, Sathiyamoorthy Mathanalagan, Asoka Gunaratne, Nimangee Mithraratne, Kaushila Thilakasiri, Chamila Pilimatalawwe, Y A Hasitha Dilhani, Marie Fernando, Kumudini Ranatunge, Loranthi Samarasinghe, Manori Vaas, Manoj Edirisooriya, Chathurani Sigera, Janaki Arumoli, Kesharie De Silva, Bimal Kudavidanage, Visanthi Pinto, Lakshman Dissanayake, Kaweesak Chittawatanarat, Napplika Kongpolprom, Udomsak Silachamroon, Prapaporn Pornsuriyasak, Tananchai Petnak, Pongsasit Singhatas, Viratch Tangsujaritvijit, Suthat Rungruanghiranya, Annop Piriyapatsom, Kanokkarn Juntaping, Konlawij Trongtrakul, Poungrat Thungtitigul, Pattraporn Tajarernmuang, Sunisa Chatmongkolchart, Rungsun Bhurayanontachai, Osaree Akaraborworn, Asma Navasakulpong, Karjbundid Surasit, Louise Thwaites, Behzad Nadjm, Dat Vu Quoc, Ha Nguyen Thi Thanh, Kinh Nguyen Van, Thuy Duong Bich, Yen Lam Minh

**Affiliations:** a Department of Intensive Care, Amsterdam University Medical Centers, Academic Medical Center, Amsterdam, Netherlands; b Laboratory of Experimental Intensive Care and Anaesthesiology, Amsterdam University Medical Centers, Academic Medical Center, Amsterdam, Netherlands; c Mahidol–Oxford Tropical Medicine Research Unit, Faculty of Tropical Medicine, Mahidol University, Bangkok, Thailand; d Section of Operative Research, Doctors with Africa, CUAMM, Padova, Italy; e Department of Intensive Care, Miulli Regional General Hospital, Acquaviva delle Fonti, Bari, Italy; f School of Public Health and Preventive Medicine, Monash University, Australian; g New Zealand Intensive Care Research Centre, Melbourne, VIC, Australia; h Department of Critical Care, Melbourne Medical School, University of Melbourne, Austin Hospital, Melbourne, Australia; i Data Analytics Research and Evaluation Centre, Austin Hospital, Melbourne, VIC, Australia; j Department of Critical Care Medicine, Hospital Israelita Albert Einstein, São Paulo, Brazil; k Department of Critical Care Medicine, Hospital das Clinicas HCFMUSP, Faculdade de Medicina, Universidade de São Paulo, São Paulo, Brazil; l Keenan Research Centre for Biomedical Science, Li Ka Shing Knowledge Institute, St Michael's Hospital, Toronto, ON, Canada; m Interdepartmental Division of Critical Care Medicine, University of Toronto, Toronto, ON, Canada; n Pulmonary Engineering Group, Department of Anaesthesiology and Intensive Care Medicine, University Hospital Carl Gustav Carus and Technical University Dresden, Dresden, Germany; o Department of Surgical Sciences and Integrated Diagnostics, San Martino Policlinico Hospital IRCCS for Oncology, University of Genoa, Genoa, Italy; p Nuffield Department of Medicine, University of Oxford, Oxford, UK; q Department of Intensive Care, University of Milan Bicocca, Monza, Italy; r Department of Medicine and Surgery, University of Milan Bicocca, Monza, Italy; s Department of Emergency and Intensive Care, San Gerardo Hospital, Monza, Italy; t Anaesthesia and Intensive Care Medicine, School of Medicine, National University of Ireland, and Galway University Hospitals Ireland, Galway, Ireland; u Regenerative Medicine Institute at CÚRAM Centre for Research in Medical Devices, National University of Ireland, and Galway University Hospitals Ireland, Galway, Ireland

## Abstract

**Background:**

Geoeconomic variations in epidemiology, the practice of ventilation, and outcome in invasively ventilated intensive care unit (ICU) patients without acute respiratory distress syndrome (ARDS) remain unexplored. In this analysis we aim to address these gaps using individual patient data of four large observational studies.

**Methods:**

In this pooled analysis we harmonised individual patient data from the ERICC, LUNG SAFE, PRoVENT, and PRoVENT-iMiC prospective observational studies, which were conducted from June, 2011, to December, 2018, in 534 ICUs in 54 countries. We used the 2016 World Bank classification to define two geoeconomic regions: middle-income countries (MICs) and high-income countries (HICs). ARDS was defined according to the Berlin criteria. Descriptive statistics were used to compare patients in MICs versus HICs. The primary outcome was the use of low tidal volume ventilation (LTVV) for the first 3 days of mechanical ventilation. Secondary outcomes were key ventilation parameters (tidal volume size, positive end-expiratory pressure, fraction of inspired oxygen, peak pressure, plateau pressure, driving pressure, and respiratory rate), patient characteristics, the risk for and actual development of acute respiratory distress syndrome after the first day of ventilation, duration of ventilation, ICU length of stay, and ICU mortality.

**Findings:**

Of the 7608 patients included in the original studies, this analysis included 3852 patients without ARDS, of whom 2345 were from MICs and 1507 were from HICs. Patients in MICs were younger, shorter and with a slightly lower body-mass index, more often had diabetes and active cancer, but less often chronic obstructive pulmonary disease and heart failure than patients from HICs. Sequential organ failure assessment scores were similar in MICs and HICs. Use of LTVV in MICs and HICs was comparable (42·4% *vs* 44·2%; absolute difference –1·69 [–9·58 to 6·11] p=0·67; data available in 3174 [82%] of 3852 patients). The median applied positive end expiratory pressure was lower in MICs than in HICs (5 [IQR 5–8] *vs* 6 [5–8] cm H_2_O; p=0·0011). ICU mortality was higher in MICs than in HICs (30·5% *vs* 19·9%; p=0·0004; adjusted effect 16·41% [95% CI 9·52–23·52]; p<0·0001) and was inversely associated with gross domestic product (adjusted odds ratio for a US$10 000 increase per capita 0·80 [95% CI 0·75–0·86]; p<0·0001).

**Interpretation:**

Despite similar disease severity and ventilation management, ICU mortality in patients without ARDS is higher in MICs than in HICs, with a strong association with country-level economic status.

**Funding:**

No funding.

## Introduction

Variations in human and structural resources in middle-income countries (MICs) might affect management of critically ill patients.^1^, ^2^, ^3^ Typical differences between MICs and high-income countries (HICs) have been described for diagnostic approaches in respiratory failure,^4^ haemodynamic management,^5^, ^6^, ^7^ and care of the ventilated patient.^1^, ^8^, ^9^ Epidemiology in critically ill patients might depend on geoeconomic status.^9^, ^10^ Non-modifiable factors such as a tropical setting and organisational factors, but also differences in disease severity and presence of comorbidities, might lead to substantial dissimilarities between MICs and HICs. Inequalities in distribution of income might further affect patients' outcomes, as has been shown before in patients with heart failure.^11^, ^12^

The large observational study to understand the global impact of severe acute respiratory failure (LUNG SAFE)^13^ investigators showed notable differences in demographics, disease severity, ventilation management, and mortality in ventilated patients with acute respiratory distress syndrome (ARDS) across geoeconomic regions, and that survival is associated with gross national income.^9^ Even when limiting the analysis to patients with mild ARDS, one in every three patients had a poor outcome.^14^ One key aspect of ventilator management in ARDS is the use of lung-protective ventilation, in particular low tidal volume ventilation (LTVV).^15^ LUNG SAFE showed that significantly more patients with ARDS received LTVV in HICs than in MICs.^9^

**Research in context**

**Evidence before this study**

We searched PubMed with the terms “mechanical ventilation, adult” [MeSH terms] AND “adult” [all fields] AND “respiratory” [all fields] OR “acute respiratory distress syndrome” [all fields] AND “geographic” [all fields] OR “country” [all fields] for articles published in any language between Jan 1, 1990, and Feb 28, 2021. We also reviewed the reference lists of publications identified by this search. We found several national and multinational studies of epidemiology, management, and outcomes related to ventilation. We also identified one secondary analysis focusing on geoeconomic variations in acute respiratory distress syndrome (ARDS). Yet, no study analysed variations across major geoeconomic groupings for ventilated patients without ARDS. The number of patients receiving invasive ventilation, however, is steadily growing in middle-income countries (MIC). It remains unclear whether shortages in resources might compromise care for critically ill patients, including ventilation in patients without ARDS.

**Added value of this study**

Our analysis of four large observational studies provides detailed information on epidemiology, important aspects of ventilation management, and outcomes in a large cohort of ventilated patients without ARDS from 54 countries. We report ventilation data over 4 consecutive days, allowing for detailed temporal insight into ventilation management. We identified notable differences in epidemiology between patients from MICs versus patients from high-income countries (HICs). Baseline severity scores and ventilation management were remarkably similar, in particular, the use of lung-protective ventilation was equally applied across both groups. Nevertheless, geoeconomic status had a strong association with mortality.

**Implications of all the available evidence**

Important regional differences exist in the demographics, but diseases severity and ventilation management are not different between MICs and HICs. Restrictions in resources do not seem to affect the ability to apply lung-protective ventilation in patients without ARDS, but heavily influence patients' outcome.

Most intensive care unit (ICU) patients receive invasive ventilation for a reason other than ARDS,^16^, ^17^, ^18^ even if the epidemiology of ventilated patients is being heavily changed by the current COVID-19 pandemic.^19^ It is uncertain whether similar differences in epidemiology, ventilation management, and outcomes exist in these patients across geoeconomic regions. The aim of the current pooled analysis using individual patient data of four large observational studies therefore was to investigate and compare the epidemiology, ventilation management, and outcomes in patients without ARDS in MICs and HICs. The main hypothesis was that use of LTVV differs between MICs and HICs.

## Methods

### Study design and participants

In this pooled analysis, individual data of patients without ARDS were extracted from the databases of four large prospective observational investigations into ventilation management in critically ill patients between June, 2011, and December, 2018: the epidemiology of respiratory insufficiency in critical care (ERICC) study (773 patients without ARDS in Brazil),^20^ LUNG SAFE (1069 patients without ARDS in 50 countries),^13^ the practice of ventilation in critically ill patients without ARDS (PRoVENT) study (1021 patients without ARDS in 16 countries),^16^ and the practice of ventilation in critically ill patients in MICs (PROVENT-iMiC) study (1315 patients without ARDS in ten countries in southeast Asia).^21^ LUNG SAFE, PRoVENT, and the PRoVENT-iMiC studies enrolled patients during a 4-week period; ERICC enrolled patients during a 2-month period. Patients diagnosed with ARDS on admission to hospital, those who received only non-invasive ventilation, and those with incomplete data were excluded. Our analysis has no separate ethics approval because it is pooling data from four approved studies, with the individual steering committees approving the use of the data. The need for patients' informed consent was waived in most centres in all four observational studies, as detailed in the original manuscripts of these studies. Detailed study methods of the four studies have been reported elsewhere.^13^, ^16^, ^20^, ^21^, ^22^

### Outcomes

The primary outcome was use of LTVV, defined as receiving ventilation with a tidal volume equal to or less than 8 mL/kg predicted bodyweight, for the first 3 days of mechanical ventilation. Secondary outcomes were tidal volume size (expressed in modus [most used value] absolute tidal volume, in mL/kg actual body weight, and in mL/kg predicted body weight), positive end-expiratory pressure, fraction of inspired oxygen, peak pressure, plateau pressure, driving pressure, and respiratory rate. Other secondary outcomes included epidemiological and clinical endpoints, such as patient characteristics, the risk for and actual development of ARDS after the first day of ventilation, duration of ventilation, ICU length of stay, and ICU mortality.

### Definitions and calculations

All datasets and definitions were harmonised before pooling the data, according to the case report forms and data dictionaries of the four studies. Participation of investigators from all four original studies was sought in the process. ARDS was defined according to the Berlin definition for ARDS.^23^ Risk of ARDS was defined by a lung injury prediction score of 4 or more.^24^ Disease severity at baseline was assessed using the sequential organ failure assessment (SOFA) score.^25^

Driving pressure was calculated by subtracting the level of positive end-expiratory pressure from the plateau pressure in volume-controlled ventilation, or from maximum inspiratory pressure in pressure-controlled ventilation, and only in patients with evidence of absence of spontaneous ventilation (defined as patients having equal set and measured respiratory rate, and not receiving ventilatory support via a spontaneous breathing mode).

Income of individual countries was assessed by the gross domestic product (GDP) per capita, a measure of a country's economic output that divides the country's national income by its total population.^26^

The 2016 World Bank countries classification was used to define two groupings: patients in ICUs in MICs and patients in ICUs in HICs.^26^

### Statistical analysis

Descriptive statistics were used to compare patients in MICs versus HICs, using frequencies and proportions for categorical variables, and medians with IQRs for continuous variables. For baseline characteristics, the groups were compared using Fisher's exact tests for categorical variables and Wilcoxon rank-sum tests for continuous variables. In all analyses, HICs were used as a reference.

All analyses were performed using multilevel (patients nested in hospitals nested in countries), mixed modelling with hospitals and countries as random effects. Ventilatory variables and parameters were compared among the groups, and absolute differences with the respective 95% CI were calculated as the absolute difference from a mixed-effect linear model considering the hospitals and countries as random effects to account for within-centre clustering. Categorical variables were compared as the risk difference from the same model.

Outcomes were compared between the two groups with the unadjusted risk difference extracted from the model described earlier. Additionally, all clinical outcomes were further compared after adjustment for prognostic factors in parsimonious models, considering the following variables, selected according to clinical relevance: age, type of admission (medical, surgical elective, surgical urgency, and trauma), active cancer, the partial pressure of arterial oxygen to the fraction of inspired oxygen ratio on the day ventilation started, total SOFA score on the day ventilation started, and an interaction between SOFA score and income group.

Cumulative distribution plots were used to plot the cumulative distribution frequency of ventilation variables on the day ventilation started, using vertical dotted lines to show cutoffs for each variable and horizontal dotted lines to indicate proportions of patients reaching the cutoffs. Cutoffs to form matrices were based on widely accepted values for each variable (8 mL/kg predicted bodyweight for tidal volume, 5 cm H_2_O for positive end-expiratory pressure, 30 cm H_2_O for plateau pressure, and 15 cm H_2_O for driving pressure).

Duration of ventilation was assessed in a competing risk model with death before extubation treated as competing risk. The results were described with the use of cumulative incidence function and reported as subdistribution hazard ratio with 95% CI estimated from a Fine-Gray model considering the cluster of the data. Duration of ventilation and ICU length of stay were censored at day 28 for this analysis.

In addition to the adjusted odds ratio for ICU mortality described in the models above, the results were also presented as the predicted mortality rate according to the baseline risk model including SOFA score at day 1 in each group (marginal effect plots).

A scatterplot was used to explore the associations between crude ICU mortality and individual country's GDP per capita. A variable life-adjusted display (VLAD) was plotted to assess the cumulative difference in survival according to income groups. VLAD is presented as the cumulative excess of survival by age, and was computed according to the difference of the expected and observed ICU mortality, with the expected mortality being derived from the baseline risk model. When a patient survived, their probability of death was added and when a patient died, their probability of death was removed. The VLAD analysis was corrected by the total number of patients in each group. The performance of the baseline risk model was assessed according to its discrimination (area under the curve) and calibration (through calibration belts). Finally, attributable fraction analysis was used to assess the proportion of ICU deaths attributable to admissions in MICs, considering the cluster of the data. Only complete case analyses were carried out.

The rate of missing data is shown in the appendix (pp 7–8) and a sensitivity analysis for the clinical outcomes considering multiple imputation for missing data was performed. Multiple imputation used chained equations considering baseline variables and outcomes, and five imputed datasets. All analyses were conducted in R (v.3.60) and a p value below 0·05 was considered statistically significant.

### Role of the funding source

There was no funding source for this study.

## Results

Of the 7608 patients included in the ERICC, LUNG SAFE, PRoVENT, and PRoVENT-iMiC studies, 3852 patients without ARDS from 534 ICUs across six continents were included in our analysis (appendix p 2). Of these patients, 2345 (61·5%) were in 27 MICs and 1507 (38·5%) were in 27 HICs. Characteristics of participating centres, economic status of the groups, and number of patients recruited per country are reported in the appendix (pp 9–10). There was a four-fold difference in median GDP per capita between MICs and HICs, with a large range of GDP among studied countries (from $US777 to $81 000). Non-academic hospitals were more common in MICs. The number of physicians per ICU was higher in HICs, but the number of nurses per bed was similar in MICs and HICs.

**Figure 1:**
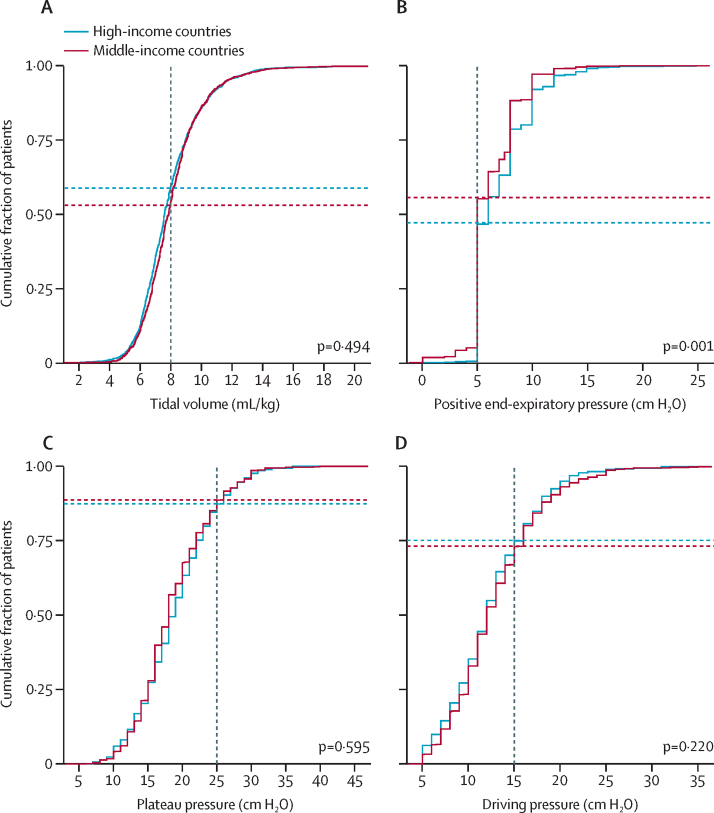
Ventilation parameters on the first day of mechanical ventilation in patients stratified by economic group Cumulative frequency distribution of tidal volume (A), positive end-expiratory pressure (B), plateau pressure (C), and driving pressure (D). Vertical dotted lines represent the cutoff for each variable and horizontal dotted lines represent the respective proportion of patients reaching each cutoff.

Patients in MICs had significantly lower age, height, and body-mass index (BMI; table 1). SOFA scores at ICU admission were not different between MICs and HICs. Patients in MICs had diabetes and active cancer more often, were more frequently classified as non-surgical patients, and more often had sepsis and non-cardiogenic shock as the reason for ICU admission. Chronic obstructive pulmonary disease and heart failure were more common in patients from HICs. Limitation of support was more frequently reported in HICs. Ventilator settings and parameters are shown in figure 1, table 2, and the appendix (p 3). Use of LTVV in the first 3 days was comparable between patients in MICs and patients in HICs (42·4% *vs* 44·2%; absolute difference –1·69% [95% CI –9·58 to 6·11]; p=0·67; table 2). Tidal volume expressed in mL/kg actual bodyweight and predicted bodyweight was not different between patients in MICs and HICs, with a lower applied absolute median tidal volume used in MICs. A comparable distribution of tidal volume was observed between the two groups (appendix p 4). The difference in applied positive end-expiratory pressure, peak and plateau pressure, driving pressure, fraction of inspired oxygen, and respiratory rate was not meaningfully different between HICs and MICs. Arterial CO_2_ pressure was lower in patients in MICs.

**Table 1:** Baseline characteristics of patients

		**Middle-income countries (n=2345)**	**High-income countries (n=1507)**	**p value**
Age, years		60 (43–72)	64 (52–75)	<0·0001
Gender				
	Female	941/2325 (40·5%)	530/1501 (35·3%)	
	Male	1384/2325 (59·5%)	971/1501 (64·7%)	0·0015
Height, cm		165 (160–170)	170 (163–177)	<0·0001
Weight, kg		70 (62–80)	76 (65–89)	<0·0001
Body-mass index, kg/m^2^		25 (22–28)	26 (23–29)	<0·0001
Type of admission		..	..	<0·0001
	Medical	1443/2321 (62·2%)	848/1496 (56·7%)	..
	Surgical elective	401/2321 (17·3%)	289/1496 (19·3%)	..
	Surgical urgency	322/2321 (13·9%)	282/1496 (18·9%)	..
	Trauma	155/2321 (6·7%)	77/1496 (5·1%)	..
Lung injury prediction score*		4 (3–6)	4 (2–6)	<0·0001
	Number at risk of ARDS	953/1508 (63·2%)	257/653 (39·4%)	<0·0001
Sequential organ failure assessment score				
	Total	7 (5–10)	7 (5–10)	0·033
	Neurological	3 (0–4)	2 (0–4)	0·070
	Renal	0 (0–2)	1 (0–3)	<0·0001
	Respiratory	2 (0–3)	2 (2–3)	<0·0001
	Haematological	1 (1–1)	0 (0–1)	<0·0001
	Liver	0 (0–0)	0 (0–1)	0·0047
	Circulatory	0 (0–3)	1 (0–2)	0·96
Comorbidities				
	Chronic obstructive pulmonary disease	207/2323 (8·9%)	294/1482 (19·8%)	<0·0001
	Diabetes	600/2324 (25·8%)	316/1489 (21·2%)	0·0014
	Chronic kidney disease	262/2324 (11·3%)	163/1490 (10·9%)	0·79
	Active cancer	322/2321 (13·9%)	169/1484 (11·4%)	0·029
	Immunosuppression	73/1095 (6·7%)	103/1490 (6·9%)	0·87
	Haematological cancer	52/2063 (2·5%)	21/854 (2·5%)	1·00
	Heart failure	227/2324 (9·8%)	190/1487 (12·8%)	0·0044
	Chronic liver failure	99/2324 (4·3%)	52/1489 (3·5%)	0·27
Risk factors for ARDS				
	Pneumonia	528/2324 (22·7%)	416/1491 (27·9%)	0·0003
	Non-pulmonary sepsis	329/1702 (19·3%)	167/1491 (11·2%)	<0·0001
	Gastric aspiration	151/2324 (6·5%)	172/1491 (11·5%)	<0·0001
	Pancreatitis	2/215 (0·9%)	17/854 (2·0%)	0·45
	Trauma	163/2344 (7·0%)	83/1507 (5·5%)	0·085
	Smoke inhalation	34/1723 (2·0%)	23/1507 (1·5%)	0·41
	Pulmonary contusion	29/1702 (1·7%)	52/1491 (3·5%)	0·0020
	Burn	2/215 (0·9%)	4/854 (0·5%)	0·77
	Pulmonary vasculitis	1/215 (0·5%)	7/854 (0·8%)	0·92
	Non-cardiogenic shock	319/2345 (13·6%)	129/1507 (8·6%)	<0·0001
	Near-drowning	3/1723 (0·2%)	1/1507 (0·1%)	0·71
	Drug overdose	4/215 (1·9%)	23/854 (2·7%)	0·65
	Transfusion-related acute lung injury	8/215 (3·7%)	26/854 (3·0%)	0·77
Limitation of support		65/1701 (3·8%)	203/1486 (13·7%)	<0·0001

Data are median (IQR) or n/N (%). ARDS=acute respiratory distress syndrome.

* Data available in 2161 (56·1%) of 3852 patients.

**Table 2:** Ventilatory parameters in the first 3 days of mechanical ventilation

	**Middle-income countries (n=2345)**	**High-income countries (n=1507)**	Absolute difference (95% CI)*	**p value**
**Primary outcome**				
Use of LTVV in first 3 days†	752/1775 (42·4%)	619/1399 (44·2%)	−1·69 (−9·58 to 6·11)	0·67
**Day 1**				
Use of LTVV†	933/1762 (53·0%)	810/1379 (58·7%)	−5·62 (−12·54 to 1·27)	0·12
Tidal volume, mL	458 (400 to 500)	495 (430 to 552)	−33·19 (−51·01 to −15·44)	0·0010
Mode	500	500	..	..
Tidal volume, mL/kg predicted bodyweight	7·9 (6·8 to 9·1)	7·6 (6·6 to 9·0)	0·10 (−0·19 to 0·39)	0·49
Tidal volume, mL/kg actual bodyweight	6·8 (5·8 to 7·7)	6·4 (5·4 to 7·8)	0·09 (−0·30 to 0·48)	0·65
PEEP, cm H_2_O	5 (5 to 8)	6 (5 to 8)	−1·04 (−1·62 to −0·46)	0·0011
FiO_2_	0·50 (0·40 to 0·60)	0·50 (0·40 to 0·60)	0·00 (−0·04 to 0·04)	0·88
Peak pressure, cm H_2_O	22 (18 to 27)	22 (18 to 27)	0·91 (−1·00 to 2·84)	0·36
Plateau pressure, cm H_2_O	18 (15 to 22)	19 (15 to 22)	−0·44 (−2·01 to 1·16)	0·59
Driving pressure, cm H_2_O	12 (10 to 16)	12 (9 to 15)	0·95 (−0·50 to 2·43)	0·22
Total respiratory rate, mpm	17 (14 to 20)	16 (14 to 20)	0·60 (−0·71 to 1·92)	0·36
Arterial pH	7·36 (7·29 to 7·42)	7·36 (7·29 to 7·43)	−0·01 (−0·02 to 0·01)	0·59
PaO_2_/FiO_2_	240 (162 to 347)	210 (150 to 278)	15·48 (−12·71 to 43·15)	0·28
PaCO_2_, mm Hg	37·0 (31·1 to 44·0)	41·0 (36·0 to 48·8)	−4·90 (−6·54 to −3·23)	<0·0001
**Day 2**				
Use of LTVV†	788/1391 (56·6)	611/1064 (57·4)	−0·70 (−7·24 to 5·67)	0·83
Tidal volume, mL	450 (400 to 500)	500 (427 to 572)	−38·66 (−57·93 to −18·92)	0·0006
Mode	500	500	..	..
Tidal volume, mL/kg predicted bodyweight	7·8 (6·8 to 8·9)	7·8 (6·7 to 9·0)	−0·04 (−0·35 to 0·27)	0·80
Tidal volume, mL/kg actual bodyweight	6·6 (5·7 to 7·6)	6·6 (5·5 to 7·8)	0·04 (−0·35 to 0·44)	0·83
PEEP, cm H_2_O	5 (5 to 8)	6 (5 to 8)	−0·97 (−1·53 to −0·41)	0·0016
FiO_2_	0·40 (0·35 to 0·50)	0·40 (0·35 to 0·50)	0·02 (−0·01 to 0·05)	0·31
Peak pressure, cm H_2_O	21 (18 to 26)	21 (17 to 27)	0·66 (−1·33 to 2·64)	0·52
Plateau pressure, cm H_2_O	18 (16 to 22)	19 (15 to 23)	−0·71 (−2·20 to 0·72)	0·35
Driving pressure, cm H_2_O	13 (10 to 16)	12 (9 to 15)	0·49 (−0·87 to 1·88)	0·50
Total respiratory rate, mpm	18 (15 to 21)	18 (15 to 22)	0·24 (−1·06 to 1·54)	0·72
Arterial pH	7·39 (7·33 to 7·44)	7·39 (7·34 to 7·44)	0·00 (−0·01 to 0·02)	0·93
PaO_2_/FiO_2_	276 (196 to 370)	236 (178 to 312)	26·69 (3·89 to 49·27)	0·027
PaCO_2_, mm Hg	36·0 (31·0 to 42·0)	40·0 (36·0 to 46·0)	−5·73 (−7·73 to −3·79)	<0·0001
**Day 3**				
Use of LTVV†	593/984 (60·3)	485/807 (60·1)	−0·90 (−8·09 to 6·05)	0·80
Tidal volume, mL	450 (400 to 500)	485 (427 to 568)	−36·71 (−57·23 to −15·31)	0·0017
Mode	500	450	..	..
Tidal volume, mL/kg predicted bodyweight	7·6 (6·7 to 8·8)	7·6 (6·5 to 9·0)	−0·07 (−0·40 to 0·26)	0·67
Tidal volume, mL/kg actual bodyweight	6·6 (5·7 to 7·6)	6·5 (5·5 to 7·7)	0·17 (−0·27 to 0·61)	0·47
PEEP, cm H_2_O	6 (5 to 8)	6 (5 to 9)	−1·13 (−1·67 to −0·59)	0·0002
FiO_2_	0·40 (0·30 to 0·50)	0·40 (0·30 to 0·50)	0·02 (−0·01 to 0·05)	0·22
Peak pressure, cm H_2_O	22 (18 to 26)	21 (17 to 26)	1·34 (−0·58 to 3·27)	0·18
Plateau pressure, cm H_2_O	18 (16 to 22)	19 (15 to 22)	0·03 (−1·50 to 1·6)	0·97
Driving pressure, cm H_2_O	12 (10 to 16)	12 (9 to 15)	0·73 (−0·56 to 2·08)	0·30
Total respiratory rate, mpm	18 (15 to 22)	18 (15 to 23)	−0·52 (−1·76 to 0·72)	0·42
Arterial pH	7·40 (7·35 to 7·45)	7·41 (7·36 to 7·45)	0·00 (−0·02 to 0·01)	0·55
PaO_2_/FiO_2_	271 (195 to 360)	237 (175 to 306)	20·88 (−1·01 to 42·35)	0·066
PaCO_2_, mm Hg	37·0 (32·0 to 43·0)	41·0 (37·0 to 46·7)	−4·78 (−6·58 to −2·97)	<0·0001

Data are median (IQR) or n/N (%). LTVV=low tidal volume ventilation. PEEP=positive end-expiratory pressure. FiO_2_=fractional concentration of oxygen in inspired air. PaO_2_=partial pressure of arterial oxygen. PaCO_2_=partial pressure of arterial carbon dioxide. mpm=movements per min.

* Absolute difference calculated from a mixed-effect linear model with study and groups as fixed effect and hospitals and country as random effect.

† Denominators show the number of patients with available tidal volume and height data in which the use of LTVV could be assessed.

Patients in MICs had a higher risk of ARDS than patients in HICs according to the lung injury prediction score (63·2% *vs* 39·4%; p=0·020; table 3). However, development of ARDS after start of ventilation, duration of ventilation, and ICU length of stay were similar in patients in MICs and in HICs. When accounting for the competing risk of death before extubation, the probability over time of extubation was similar in patients from MICs versus HICs (appendix p 5).

**Table 3:** Clinical outcomes according to the economic group

	**Middle-income countries (n=2345)**	**High-income countries (n=1507)**	Unadjusted effect (95% CI)*	**p value**	**Adjusted effect (95% CI)**†	**p value**
Patients at risk of ARDS	953/1508 (63·2%)	257/653 (39·4%)	17·88 (4·32 to 31·49)‡	0·020	14·26 (3·30 to 25·25)‡	0·023
Development of ARDS during follow-up	184/2281 (8·1%)	151/1423 (10·6%)	−3·78 (−8·35 to 0·73)‡	0·11	−2·96 (−7·39 to 1·36)‡	0·19
Duration of ventilation, days	3·0 (1·0 to 7·0%)	4·0 (2·0 to 10·0%)	0·91 (0·80 to 1·05)§	0·20	0·93 (0·81 to 1·06)§	0·26
ICU length of stay, days	6·0 (2·0 to 12·0%)	7·0 (3·0 to 14·0%)	−1·00 (−4·31 to 2·26)¶	0·55	0·08 (−3·28 to 3·37)¶	0·96
ICU mortality	684/2243 (30·5%)	283/1419 (19·9%)	15·67 (7·98 to 23·60)‡	<0·0004	16·41 (9·52 to 23·52)‡	<0·0001

Data are median (IQR) or n/N (%). ARDS=acute respiratory distress syndrome. ICU=intensive care unit.

* Unadjusted effect calculated from models with group as fixed effect and hospitals and countries as random effect.

† Adjusted effect calculated from models with group as fixed effect, hospitals and countries as random effect, and adjusted for age, type of admission, active cancer, partial pressure of arterial oxygen to the fraction of inspired oxygen ratio at day 1, total sequential organ failure assessment (SOFA) score at day 1, and an interaction between SOFA and the group.

‡ Effect estimate is risk difference from a mixed-effect model.

§ Effect estimate is subdistribution hazard ratio from a Fine-Gray model considering the cluster of the data.

¶ Effect estimate is mean difference from a mixed-effect model.

**Figure 2:**
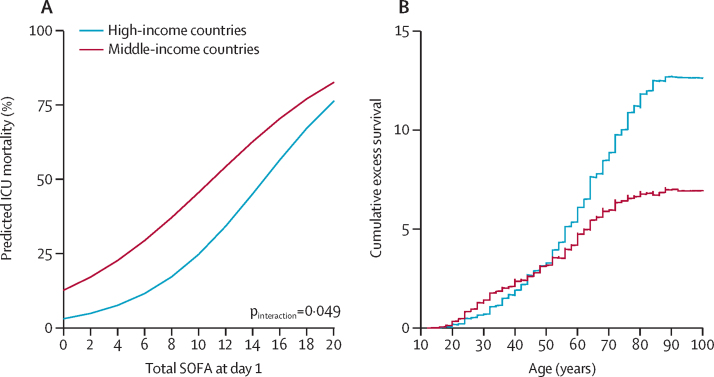
Marginal effect plot (A) showing the predicted mortality according to the SOFA score at day 1 and variable life-adjusted display (B) to assess cumulative excess survival according to income groups ICU=intensive care unit. SOFA=sequential organ failure assessment.

**Figure 3:**
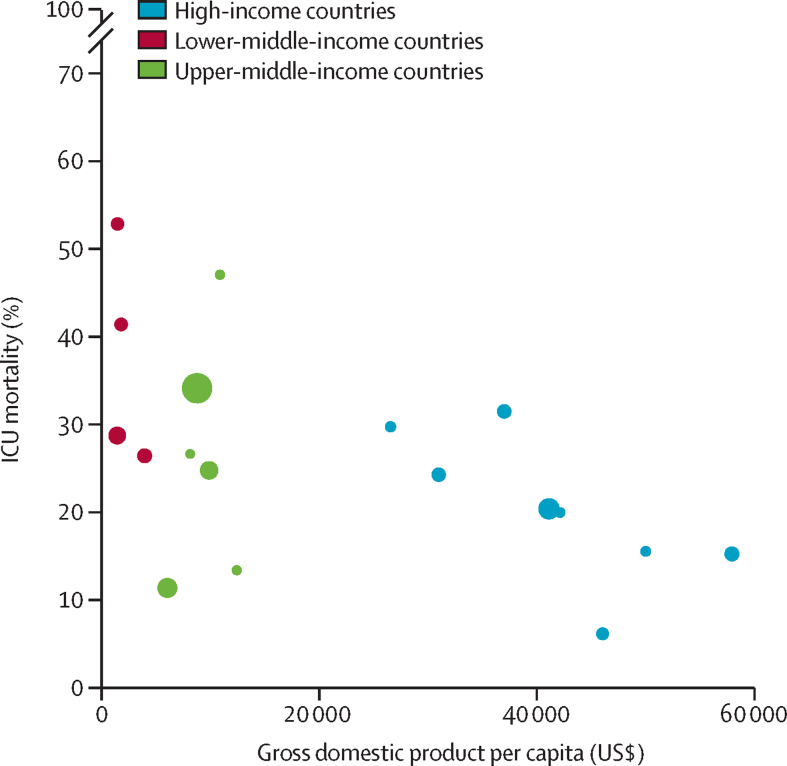
Scatter plot exploring the association between crude intensive care unit mortality and gross domestic product per capita Each circle represents a country. The size of the circle reflects the number of enrolled patients in the country (appendix pp 8–9). Middle-income countries were further divided into lower-middle-income (red) and upper-middle-income countries (green). Countries that recruited fewer than 50 patients were excluded.

ICU mortality was approximately 1·5-fold higher in patients from MICs than from HICs. The higher probability of death in patients in MICs was particularly pronounced in patients with lower SOFA scores (figure 2A). In patients older than 50 years, cumulative excess survival was lower in MICs, and the gap increased with patient age (figure 2B; appendix p 5). The proportion of deaths attributable to admissions in MICs was 22·1% (95% CI 14·3–29·9; p<0·0001). Crude ICU mortality was inversely associated with GDP per capita (adjusted odds ratio for a US$10 000 increase in GDP per capita 0·80 [95% CI 0·75–0·86]; p<0·0001; figure 3). The sensitivity analysis after multiple imputation did not change the findings (appendix p 12). Characteristics and outcomes of patients with and without missing data in the primary outcome were comparable (appendix pp 13–14).

## Discussion

The results of this pooled analysis of four large observational studies in invasively ventilated patients without ARDS can be summarised as follows: (1) the practice of ventilation, in particular the use of LTVV, does not differ between MICs and HICs; (2) in MICs more patients are at risk of ARDS but development of ARDS is comparable to that in HICs; (3) there are remarkable baseline differences between MICs and HICs regarding age, height, and comorbidities, but disease severity at ICU admission, ICU length of stay, and timing of extubation are similar in MICs and HICs; and (5) ICU mortality is significantly higher in MICs, with a strong association between country-level economic status and survival.

Strengths of this analysis are the availability of individual patient data of large groups of patients captured in more than 500 ICUs worldwide. All four studies had a prospective design, and included measures to limit selection and observation bias. The short time span between the studies minimises the risk of effects of large changes in processes of care. The studies had several endpoints in common, which allowed for reliable merging of ventilation data. The statistical analysis plan for this meta-analysis was predefined and strictly followed.

MICs host a majority of the 14 million patients potentially in need of invasive ventilation each year.^18^ The hypothesis that limitations in resources hamper physicians in MICs in applying LTVV^27^, ^28^, ^29^ is rejected by the current analysis. This finding differs from the results of a 2017 study in patients with ARDS, whereby fewer patients in MICs received a tidal volume of less than 8mL/kg predicted bodyweight than in non-European HICs, although the differences were small.^9^ One salient finding of our study is that half of patients without ARDS did not receive LTVV. The benefit of LTVV has been clearly demonstrated for patients with ARDS, and there is potential benefit for patients without ARDS.^30^, ^31^ A 2018 randomised trial did not show a difference in ventilator-free days and alive at day 28 when comparing a low versus intermediate tidal volume strategy in patients without ARDS.^17^ Yet because physician recognition of ARDS remains challenging,^13^ and because patients who do not fulfil the current definition for ARDS still might have lung injury, targeting a low tidal volume in all patients has been suggested—ie, irrespective of the diagnosis of ARDS.^32^

A large difference in height was found among patients being ventilated in MICs versus HICs. This finding is important, as shorter patients and especially women with ARDS have been shown to be at higher risk of receiving higher tidal volume, although geoeconomic variations were not shown to modify the relationship between sex and mortality.^33^, ^34^ Beyond sex, variations in height depend on ethnicity-based anthropometric differences, nutritional status, and other population-specific characteristics. Our findings in patients without ARDS suggest that smaller absolute tidal volumes seem to be applied well in MICs, thus not affecting the use of lung-protective ventilation. Further research is needed to explore whether female patients without ARDS are at higher risk of injurious ventilation and poorer outcomes than men.

The difference in risk for ARDS in MICs and HICs seems not to be in line with the comparable proportion of patients actually developing ARDS in the two groupings. The lung injury prediction score performs poorly in predicting ARDS, as also suggested by one validation study^24^ and subsequent investigations.^16^, ^21^, ^35^ However, we cannot exclude the possibility that ARDS was underdiagnosed in ICUs in MICs. Although, physicians in MICs seem to recognise ARDS equally well or even better than those in HICs,^9^, ^36^ some ICUs might not have the resources to apply the Berlin definition for ARDS such as chest x-ray imaging and blood gas analysis.^4^ In the PRoVENT-iMIC study performed in Asia, which reported availability of diagnostic tools, x-ray apparatuses and blood gas analysers were available for over 90% of centres, although the resources were often shared with the hospital and not dedicated to the ICU.^21^ LUNG SAFE was the only study to use an additional algorithm-based tool to identify patients who fulfilled the Berlin definition of ARDS.^13^

Although notable differences were found in demographics and comorbidities, there was neither a difference in diseases severity on ICU admission, nor in the practice of ventilation, between HICs and MICs. Thus, the higher ICU mortality in MICs requires alternative explanations. The SOFA score used to assess severity is predictive for mortality,^25^ but this process might need refinement in MICs. Beyond ventilator settings, there are additional ventilation management factors to consider.^1^ For instance, differences in infection prevention and control policies might influence the rates of health-care-associated infections. Ventilator-associated pneumonia occurs more frequently and is more often caused by antimicrobial resistant pathogens in ICUs in Asia than in high-income settings.^37^ Airway care also affects ventilation management, and acute endotracheal tube occlusions were reported in 38% of ventilated ICU patients in a 2015 Indian study.^38^

There is a wide range of potential factors at different levels of the care process that can contribute to the observed higher case fatality of mechanically ventilated patients in MICs. These factors include shortages in human and structural resources in low-income and MICs, limited use of treatment protocols, suboptimal ICU processes organisation, higher prevalence of antimicrobial drug resistance, higher bed-to-nurse ratio, or not having a daily plan of care review in place.^39^, ^40^ However, the quantitative contribution of these factors has not been well characterised.

Excess mortality in MICs increased with patient age, possibly reflecting a struggle in treating older patients who might have more comorbidities and reduced functional capacity. A 2018 study from Kenya showed high mortality despite the use of advanced therapies, with increased odds for mortality observed in age groups older than 35 years.^2^ A lack of resources did not fully explain the differences in outcomes in ARDS patients,^9^ and unmeasured social determinants might influence patients' prognosis.^41^ Also, physicians in MICs might be more likely to accommodate families' requests to prematurely stop critical care on financial grounds, although our data did not corroborate this tendencey.^40^, ^42^ Differences in primary diagnosis and reason of admission to ICU might play a role—ie, more postoperative admissions in HICs than in MICs. Research agendas should explore these and other socioeconomic, demographic, genetic, and causative context-specific factors that interact to affect ICU mortality.

This analysis has limitations. The individual studies enrolled convenience samples, but with an over-representation of academic centres or teaching hospitals. This over-representation might affect the generalisability of the study findings to the wider MIC setting, because large public hospitals, known for their large patient load in relation to available medical staff and potential worse adherence to guidelines, were under-represented. The studies had no access to patients' source data; hence some degree of selection and reporting biases cannot be excluded. As with other analyses that pool data from large observational studies, residual confounding cannot be excluded. Missing tidal volumes or height data in some patients hampered assessing the use of LTVV. The number of patients recruited per country and ICU was highly heterogeneous. No data were available for pivotal characteristics such as functional status on admission; similarly, the SOFA score was the only severity score transversally available across the four studies, and thus described comorbidities are not included in the severity assessment. Also, only one low-income country was represented, which might reflect the resource-dense nature of ICU care.^9^ Finally, economic status could only be analysed at a national level, and not at patient or family level.

In summary, there is no geoeconomic variation in the application of lung-protective ventilation in ICU patients without ARDS. A strong association, however, exists between country-level economic status and severity-adjusted survival of invasively ventilated patients without ARDS. Further research is needed to identify which factors explain the higher mortality in MICs.

## Data sharing

A deidentified dataset will be made available upon request to the corresponding author at least 1 year after the publication of this study. The request must include a statistical analysis plan.

## Declaration of interests

We declare no competing interests.
