# Supplementary material for: Geoeconomic variations in epidemiology, ventilation management, and outcomes in invasively ventilated intensive care unit patients without acute respiratory distress syndrome: a pooled analysis of four observational studies
Source: Lancet Glob Health. 2021 Dec 13;10(2):e227–35. doi: 10.1016/S2214-109X(21)00485-X (PMC8766316; doi:10.1016/S2214-109X(21)00485-X)
Supplement: Supplementary appendix [file mmc1.pdf]

THE LANCET
Global Health

Supplementary appendix

This appendix formed part of the original submission and has been peer reviewed. We post it as supplied by the authors.

Supplement to: Pisani L, Geke Algera A, Serpa Neto A, et al. Geoeconomic variations in epidemiology, ventilation management, and outcomes in invasively ventilated intensive care unit patients without acute respiratory distress syndrome: a pooled analysis of four observational studies. *Lancet Glob Health* 2021; published online Dec 13. https://doi.org/10.1016/S2214-109X(21)00485-X.

Electronic Supplement to

Geo–Economic Variations in Epidemiology, Ventilation Management and Outcome in invasively ventilated ICU patients without ARDS – a pooled analysis of 4 observational studies

ADDITIONAL RESULTS 2
eFigure 1. Patient flow 2
eFigure 2 - Ventilatory parameters in the first 3 days of mechanical ventilation in patients stratified by economic group 3
eFigure 3 - Histogram of tidal volume stratified by economic group 4
eFigure 4. Probability of discontinuing mechanical ventilation when accounting for the competing risk of death before extubation 5
eFigure 4 - Performance of baseline risk model 6
eTable 1 - Rate of missing data 7
eTable 2 - Numbers of patients and ICUs in each country 9
eTable 3 - Characteristics of the participating ICUs, economic indices of geo–economic groups and numbers of patients enrolled by country 11
eTable 4 – Clinical outcomes according to the economic group after multiple imputation* 12
eTable 5 - Baseline characteristics and clinical outcomes according to the missing data in tidal volume per predicted body weight 13
LIST OF INVESTIGATORS FROM INCLUDED STUDIES 15
FUNDING OF INDIVIDUAL STUDIES 27

ADDITIONAL RESULTS

eFigure 1. Patient flow

Abbreviations: ERICC, 'Epidemiology of Respiratory Insufficiency in Critical Care'; LUNG SAFE, Large observational study to Understand the Global impact of Severe Acute respiratory Failure; PRoVENT, Practice of VENTilation in critically ill patients without ARDS study; PRoVENT–iMiC, Practice of VENTilation in critically ill patients in middle income countries study; ARDS, acute respiratory distress syndrome; NIV, non–invasive ventilation; LMICs, low– and middle–income countries; HICs, high–income countries

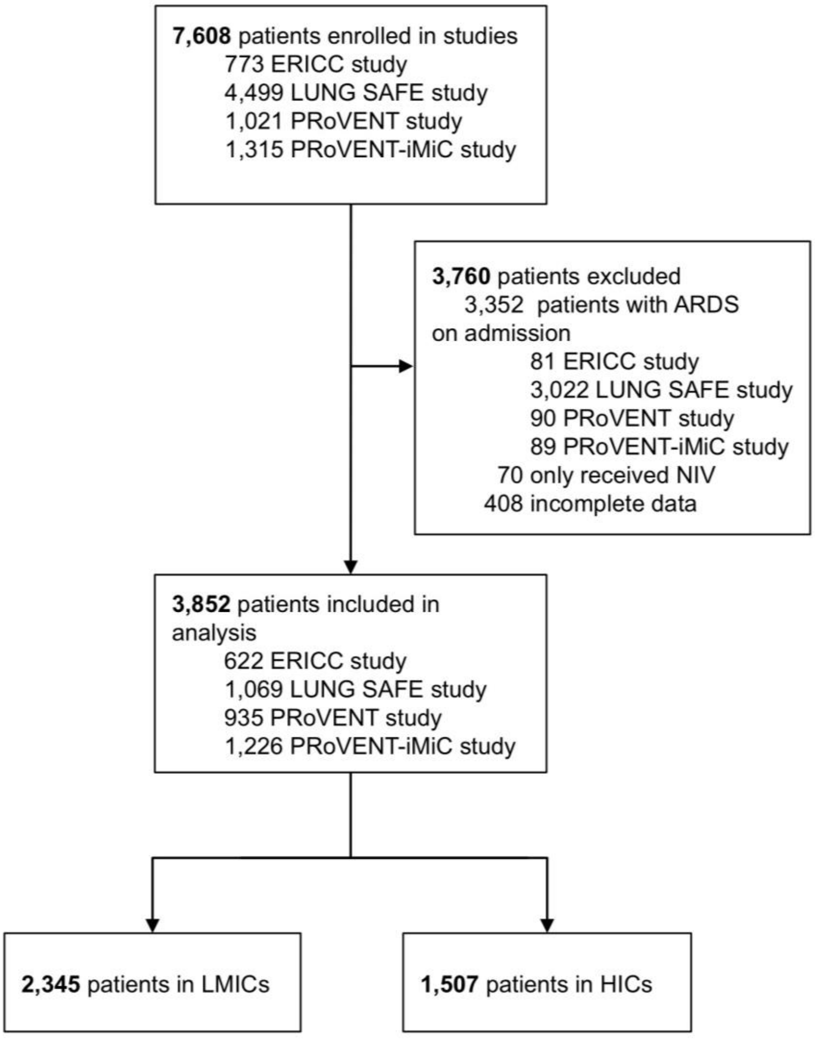

eFigure 2 - Ventilatory parameters in the first 3 days of mechanical ventilation in patients stratified by economic group
Lines are means and 95% confidence intervals. P value is for group and time–group interaction.
Abbreviations: V_T, tidal volume; PBW, predicted body weight; PEEP, positive end–expiratory pressure; Ppeak, peak pressure; FiO_2, fraction of inspired oxygen; Pplat, plateau pressure; HIC, high–income countries; MIC, middle–income countries

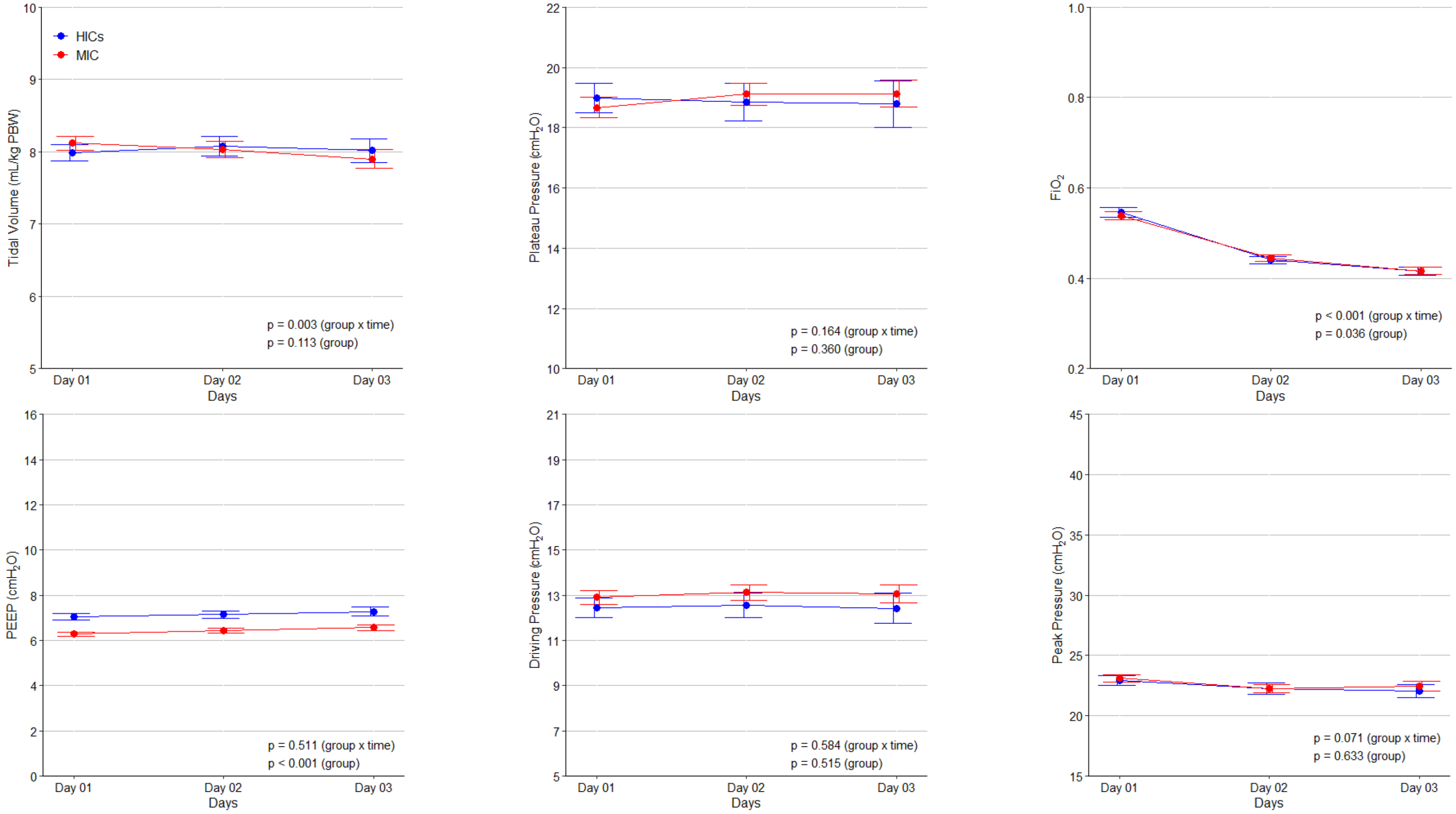

eFigure 3 - Histogram of tidal volume stratified by economic group

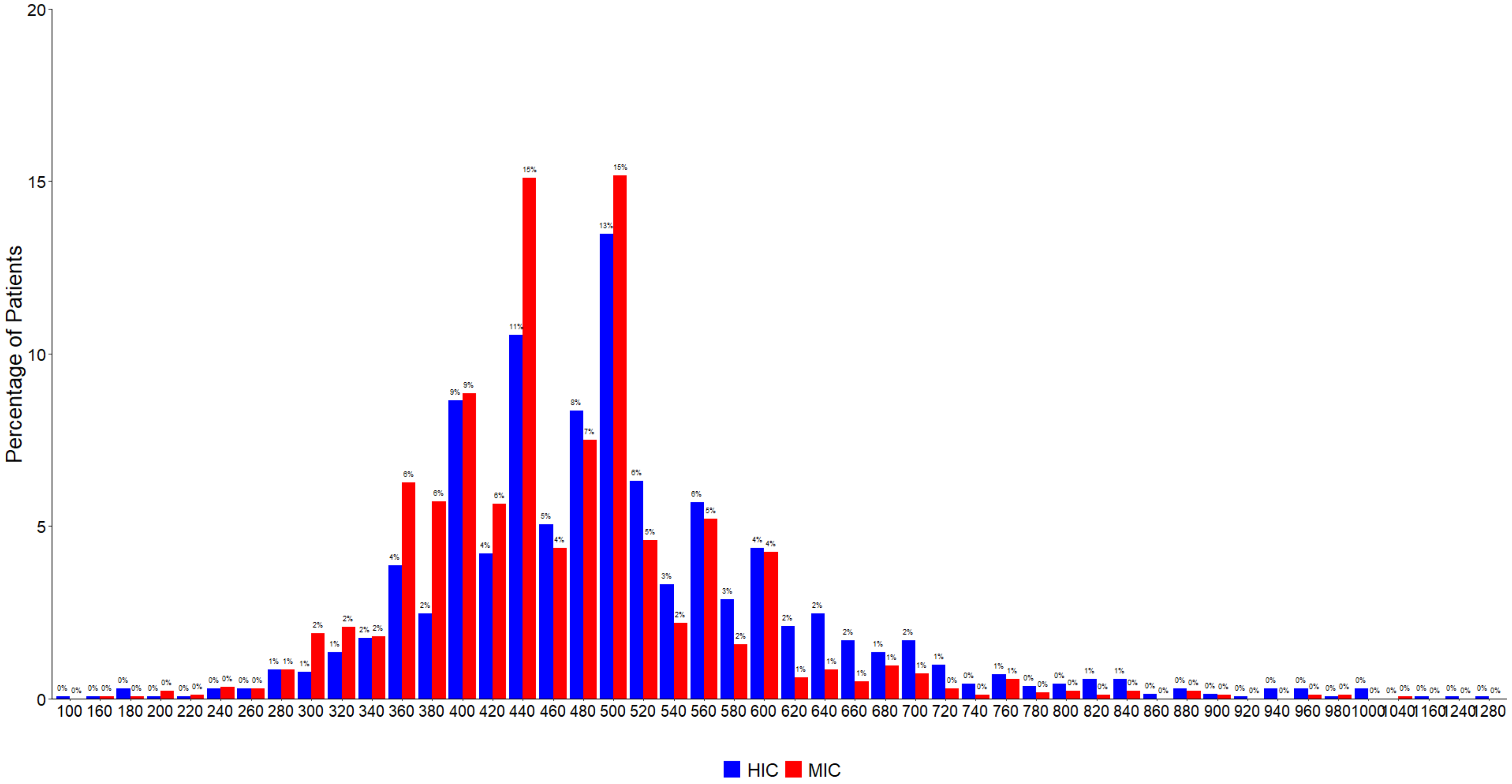

eFigure 4. Probability of discontinuing mechanical ventilation when accounting for the competing risk of death before extubation

Estimates are shown as hazard ratio (95% confidence intervals). The cumulative incidence function curves estimate the instantaneous probability over time of *extubation* (shown in continuous lines) when accounting for the risk set attrition due to the occurrence of the competing risk (*death before extubation*, shown as dotted lines). The 'Fine and Gray' proportional subdistribution hazards analysis was used to model the subdistribution hazard and derive P-values.

Abbreviations: HIC, high–income countries; MIC, middle–income countries

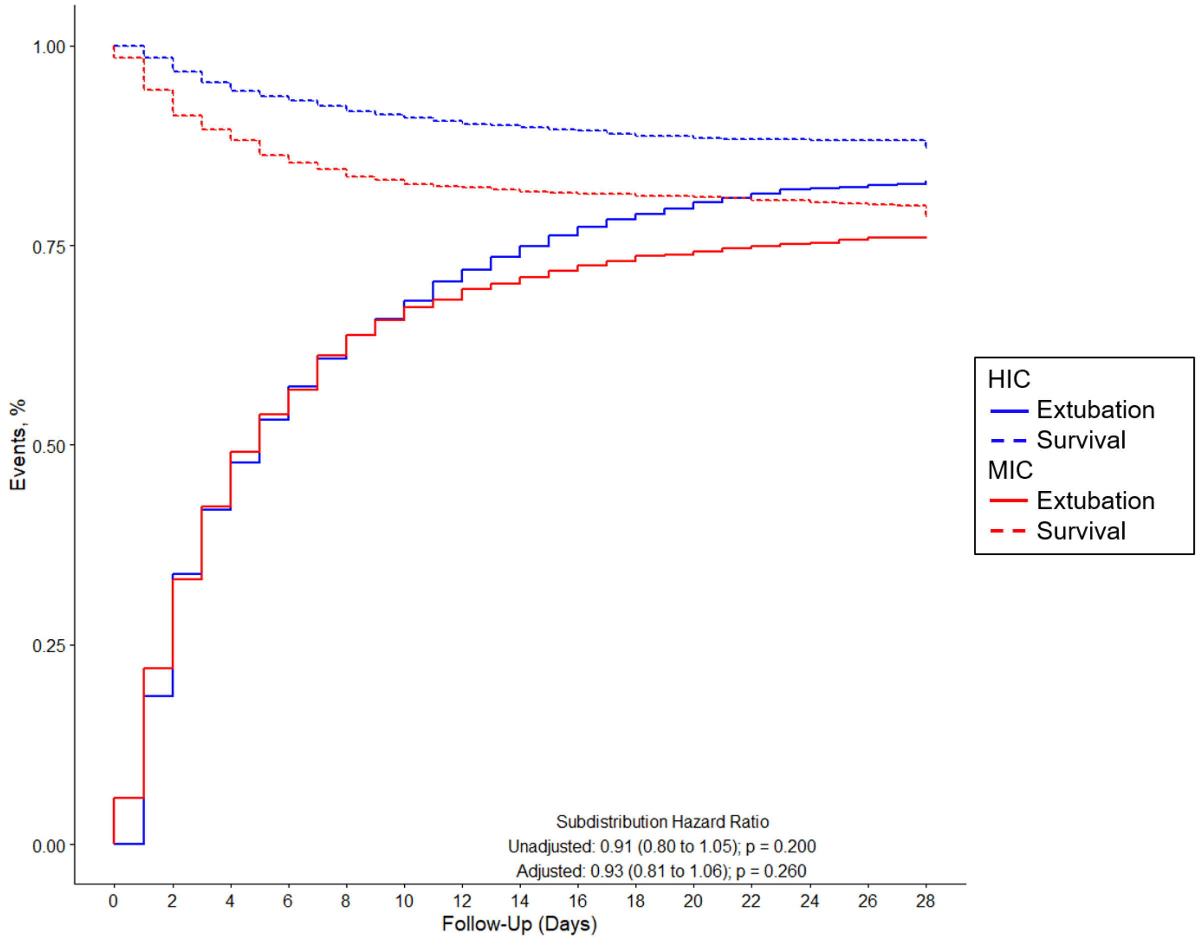

eFigure 4 - Performance of baseline risk model

The performance was assessed through area under the curve for discrimination (panel A) and calibration belts (panel B).

Abbreviations: AUC, area under the curve.

A

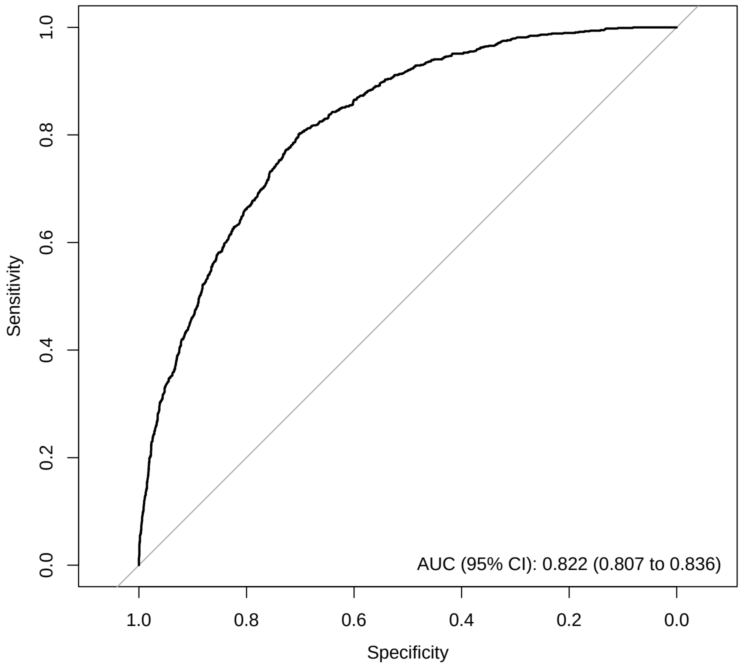

B

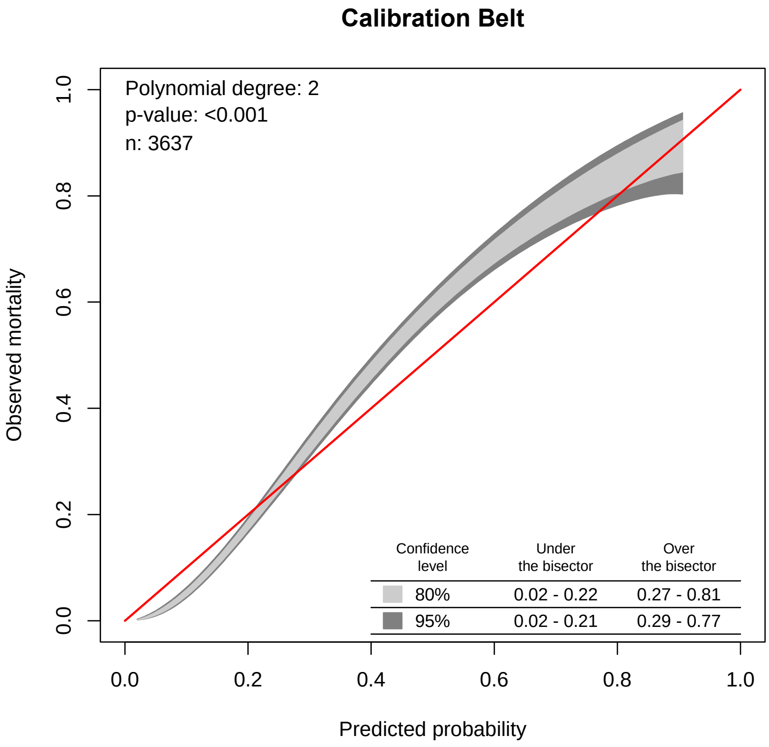

eTable 1 - Rate of missing data

	Overall (*n* = 3,852)	MIC (*n* = 2345)	HICs (*n* = 1507)
Age	26 (0.7)	20 (0.9)	6 (0.4)
Gender	26 (0.7)	20 (0.9)	6 (0.4)
Height	137 (3.6)	80 (3.4)	57 (3.8)
Weight	1360 (35.3)	1290 (55)	70 (4.6)
BMI	1408 (36.6)	1300 (55.4)	108 (7.2)
Type admission	35 (0.9)	24 (1)	11 (0.7)
LIPS	1691 (43.9)	837 (35.7)	854 (56.7)
Total SOFA	105 (2.7)	46 (2)	59 (3.9)
COPD	47 (1.2)	22 (0.9)	25 (1.7)
Diabetes mellitus	39 (1)	21 (0.9)	18 (1.2)
Chronic kidney disease	38 (1)	21 (0.9)	17 (1.1)
Active cancer	47 (1.2)	24 (1)	23 (1.5)
Immunosuppression	1267 (32.9)	1250 (53.3)	17 (1.1)
Hematologic cancer	935 (24.3)	282 (12)	653 (43.3)
Heart failure	41 (1.1)	21 (0.9)	20 (1.3)
Chronic liver failure	39 (1)	21 (0.9)	18 (1.2)
Pneumonia	37 (1)	21 (0.9)	16 (1.1)
Non–pulmonary sepsis	659 (17.1)	643 (27.4)	16 (1.1)
Gastric aspiration	37 (1)	21 (0.9)	16 (1.1)
Pancreatitis	2783 (72.2)	2130 (90.8)	653 (43.3)
Trauma	1 (0)	1 (0)	0 (0)
Smoke inhalation	622 (16.1)	622 (26.5)	0 (0)
Pulmonary contusion	659 (17.1)	643 (27.4)	16 (1.1)
Burn	2783 (72.2)	2130 (90.8)	653 (43.3)
Pulmonary vasculitis	2783 (72.2)	2130 (90.8)	653 (43.3)
Non–cardiogenic shock	0 (0)	0 (0)	0 (0)
Near–drowning	622 (16.1)	622 (26.5)	0 (0)
Drug overdose	2783 (72.2)	2130 (90.8)	653 (43.3)
TRALI	2783 (72.2)	2130 (90.8)	653 (43.3)
Limitation of support	665 (17.3)	644 (27.5)	21 (1.4)
Use of LTVV first 3 days	678 (17.6)	570 (24.3)	108 (7.2)
Use of LTVV on day 1	711 (18.5)	583 (24.9)	128 (8.5)
Tidal volume on day 1	641 (16.6)	558 (23.8)	83 (5.5)
PEEP on day 1	304 (7.9)	246 (10.5)	58 (3.8)
FiO_2 on day 1	298 (7.7)	248 (10.6)	50 (3.3)
Peak pressure on day 1	549 (14.3)	421 (18)	128 (8.5)
Plateau pressure on day 1	2416 (62.7)	1376 (58.7)	1040 (69)
Driving pressure on day 1	2475 (64.3)	1401 (59.7)	1074 (71.3)
Respiratory rate on day 1	392 (10.2)	330 (14.1)	62 (4.1)
pH on day 1	421 (10.9)	329 (14)	92 (6.1)

eTable 1 - Rate of missing data

	**Overall (*n* = 3,852)**	**MIC (*n* = 2345)**	**HICs (*n* = 1507)**
PaO_2 / FiO_2 on day 1	665 (17.3)	557 (23.8)	108 (7.2)
$PaCO_2$ on day 1	442 (11.5)	338 (14.4)	104 (6.9)
Patient at risk for ARDS	1691 (43.9)	837 (35.7)	854 (56.7)
Development of ARDS during follow–up	148 (3.8)	64 (2.7)	84 (5.6)
Duration of ventilation, days	190 (4.9)	102 (4.3)	88 (5.8)
ICU length of stay, days	474 (12.3)	274 (11.7)	200 (13.3)
ICU mortality	214 (5.6)	105 (4.5)	109 (7.2)

Data are N (%)

MIC, middle–income countries; HIC, high–income countries; BMI: body mass index; LIPS: Lung Injury Prediction Score; ARDS: acute respiratory distress syndrome; SOFA: Sequential Organ Failure Assessment; COPD: chronic obstructive pulmonary disease; TRALI: transfusion–related acute lung injury

eTable 2 - Numbers of patients and ICUs in each country

	MIC (*n* = 2,345)		HIC (*n* = 1,507)	
	**Number of ICUs**	**Number of patients**	**Number of ICUs**	**Number of patients**
Argentina	13	38 (1.6)	--	--
Bangladesh	6	123 (5.2)	--	--
Brazil	61	720 (30.7)	--	--
China	26	60 (2.6)	--	--
Colombia	1	3 (0.1)	--	--
Costa Rica	1	3 (0.1)	--	--
Croatia	6	83 (3.5)	--	--
India	11	128 (5.5)	--	--
Iran	4	21 (0.9)	--	--
Kosovo	1	6 (0.3)	--	--
Malaysia	4	247 (10.5)	--	--
Maldives	1	3 (0.1)	--	--
Mexico	5	17 (0.7)	--	--
Morocco	1	1 (0.0)	--	--
Nepal	1	18 (0.8)	--	--
Pakistan	10	232 (9.9)	--	--
Peru	1	1 (0.0)	--	--
Philippines	1	1 (0.0)	--	--
Romania	2	8 (0.3)	--	--
Russian Federation	1	4 (0.2)	--	--
Serbia	3	7 (0.3)	--	--
South Africa	1	7 (0.3)	--	--
Sri Lanka	10	159 (6.8)	--	--
Thailand	13	290 (12.4)	--	--
Tunisia	4	8 (0.3)	--	--
Turkey	19	119 (5.1)	--	--
Vietnam	2	38 (1.6)	--	--
Australia	--	--	13	79 (5.2)
Austria	--	--	1	13 (0.9)
Belgium	--	--	9	41 (2.7)
Brunei Darussalam	--	--	1	5 (0.3)
Canada	--	--	5	21 (1.4)
Chile	--	--	5	12 (0.8)
Czech Republic	--	--	3	23 (1.5)
Denmark	--	--	2	6 (0.4)
France	--	--	35	146 (9.7)
Germany	--	--	4	83 (5.5)
Greece	--	--	2	4 (0.3)

eTable 2 - Numbers of patients and ICUs in each country

	**MIC (*n* = 2,345)**		**HIC (*n* = 1,507)**	
	**Number of ICUs**	**Number of patients**	**Number of ICUs**	**Number of patients**
Ireland	--	--	7	40 (2.7)
Italy	--	--	32	158 (10.5)
Japan	--	--	21	36 (2.4)
Latvia	--	--	1	1 (0.1)
Netherlands	--	--	8	117 (7.8)
New Zealand	--	--	5	10 (0.7)
Norway	--	--	2	11 (0.7)
Poland	--	--	1	2 (0.1)
Portugal	--	--	9	28 (1.9)
Saudi Arabia	--	--	3	7 (0.5)
Spain	--	--	30	84 (5.6)
Sweden	--	--	8	18 (1.2)
Switzerland	--	--	2	9 (0.6)
United Kingdom	--	--	78	373 (24.8)
United States	--	--	32	158 (10.5)
Uruguay	--	--	6	22 (1.5)

Data are N (%)
MIC, middle–income countries; HIC, high–income countries;

eTable 3 - Characteristics of the participating ICUs, economic indices of geo–economic groups and numbers of patients enrolled by country

	MIC (*n* = 2,345)	HIC (*n* = 1,507)	*p* value
**ICU organization**			
Number of units	209 (39.1)	325 (60.9)	---
Non–academic hospitals	682 (33.5)	212 (25.2)	< 0.001
Hospital beds	900 (500 – 1350)	718 (530 – 1000)	< 0.001
ICU beds per unit	16 (10 – 30)	18 (12 – 26)	0.002
Staff physicians per unit	2 (1 – 3)	3 (2 – 5)	< 0.001
Nurses per unit	10 (8 – 15)	10 (6 – 17)	0.004
Nurse-to-bed ratio			< 0.001
1:1	98 / 681 (14.4)	712 / 1,364 (52.2)	
1:2	353 / 681 (51.8)	403 / 1,364 (29.5)	
1:3	178 / 681 (26.1)	156 / 1,364 (11.4)	
1:4 or more	52 / 681 (7.6)	93 / 1,364 (6.8)	
**GDP per capita, US–dollars**	8,713 (3,886 – 8,713)	41,074 (36,962 – 46,008)	< 0.001

Data are median (quartile 25% – quartile 75%) or N (%)
ICU: intensive care unit; GDP, gross domestic product; MIC, low– and middle–income countries; HIC, high–income countries.

eTable 4 – Clinical outcomes according to the economic group after multiple imputation*

	**LMICs (*n* = 2345)**	**HICs (*n* = 1507)**	**Adjusted Effect (95% confidence interval)****	***p* value**
Patient at risk for ARDS	953 (63.2)	257 (39.4)	20.6 (9.3 to 31.9)[a]	< 0.001
Development of ARDS during follow–up	184 (8.1)	151 (10.6)	-3.2 (-7.4 to 1.0)[a]	0.137
Duration of ventilation, days	3.0 (1.0 – 7.0)	4.0 (2.0 – 10.0)	-1.0 (-3.3 to 1.3)[b]	0.397
ICU length of stay, days	6.0 (2.0 – 12.0)	7.0 (3.0 – 14.0)	-1.0 (-3.8 to 1.8)[b]	0.480
ICU mortality	684 (30.5)	283 (19.9)	16.8 (10.3 to 23.3)[a]	< 0.001

Data are median (quartile 25% – quartile 75%) or N (%)
MIC, middle–income countries; HIC, high–income countries; ARDS: acute respiratory distress syndrome; ICU intensive care unit.
* Multiple imputation by chained equations considering baseline variables and outcomes, and five imputed datasets.
** Adjusted effect calculated from models with group as fixed effect, hospitals and countries as random effect, and adjusted for age, type of admission, active cancer, PaO_2/FiO_2 at day 1, total SOFA at day 1 and an interaction between SOFA and the group
[a]effect estimate is risk difference from a mixed–effect model
[b]effect estimate is mean difference from a mixed–effect model

eTable 5 - Baseline characteristics and clinical outcomes according to the missing data in tidal volume per predicted body weight

	No Missing			Missing		
	**MIC (*n* = 1,762)**	**HIC (*n* = 1,379)**	***p* value**	**MIC (*n* = 583)**	**HIC (*n* = 128)**	***p* value**
Age, years	59 (43 - 70)	64 (51 - 75)	< 0.001	62 (43 - 76)	65 (56 - 75)	0.147
Male gender	1058 (60.0)	900 (65.3)	0.003	326 (57.9)	71 (57.7)	0.999
Height, cm	165 (158 - 170)	170 (163 - 177)	< 0.001	167 (160 - 175)	170 (158 - 175)	0.290
Weight, kg	70 (63 - 80)	76.0 (65.0 - 89.2)	< 0.001	70 (60 - 80)	78 (66 - 86)	< 0.001
BMI, kg/m^2	24.8 (22.5 - 27.8)	25.8 (23.0 - 29.4)	< 0.001	24.7 (22.7 - 27.8)	26.4 (22.9 - 30.7)	0.021
**Type of admission**			< 0.001			0.002
Medical	1077 (61.2)	781 (56.7)		366 (65.1)	67 (56.8)	
Surgical elective	354 (20.1)	267 (19.4)		47 (8.4)	22 (18.6)	
Surgical urgency	231 (13.1)	259 (18.8)		91 (16.2)	23 (19.5)	
Trauma	97 (5.5)	71 (5.2)		58 (10.3)	6 (5.1)	
**LIPS**[#]	4 (3 - 6)	3 (2 - 5)	< 0.001	3 (0 - 5)	1 (0 - 3)	0.149
At risk of ARDS	922 (63.9)	239 (41.4)	< 0.001	31 (47.7)	18 (23.7)	0.005
**Total SOFA**	7 (5 - 10)	7 (5 - 10)	0.002	8 (5 - 10)	7 (4 - 10)	0.200
Neurological	3 (0 - 4)	2 (0 - 4)	0.043	2 (0 - 4)	3 (1 - 4)	0.515
Renal	0 (0 - 2)	1 (0 - 3)	< 0.001	0.50 (0 - 3)	0 (0 - 2)	0.393
Respiratory	2 (0 - 3)	2 (2 - 3)	< 0.001	2 (0 - 2)	2 (2 - 3)	0.016
Hematological	1 (1 - 1)	0 (0 - 1)	< 0.001	1 (0 - 1)	0 (0 - 1)	0.069
Liver	0 (0 - 0)	0 (0 - 1)	0.012	0 (0 - 0)	0 (0 - 1)	0.188
Circulatory	0 (0 - 3)	1 (0 - 2)	0.889	0 (0 - 2)	0 (0 - 2)	0.917
**Co–morbidities**						
COPD	154 (8.7)	275 (20.0)	< 0.001	53 (9.4)	19 (17.4)	0.021
Diabetes mellitus	484 (27.5)	301 (21.9)	< 0.001	116 (20.6)	15 (13.2)	0.087
Chronic kidney disease	215 (12.2)	157 (11.4)	0.527	47 (8.4)	6 (5.3)	0.363
Active cancer	218 (12.4)	154 (11.2)	0.347	104 (18.5)	15 (13.3)	0.232
Immunosuppression	53 (9.6)	96 (7.0)	0.062	20 (3.7)	7 (6.1)	0.344
Hematologic cancer	28 (1.8)	19 (2.4)	0.471	24 (4.5)	2 (3.8)	0.999
Heart failure	183 (10.4)	182 (13.2)	0.015	44 (7.8)	8 (7.1)	0.937
Chronic liver failure	82 (4.7)	47 (3.4)	0.100	17 (3.0)	5 (4.4)	0.635
**Risk factors for ARDS**						
Pneumonia	388 (22.0)	387 (28.1)	< 0.001	140 (24.9)	29 (25.9)	0.921
Non–pulmonary sepsis	318 (19.3)	158 (11.5)	< 0.001	11 (19.6)	9 (8.0)	0.053
Gastric aspiration	130 (7.4)	161 (11.7)	< 0.001	21 (3.7)	11 (9.8)	0.012
Pancreatitis	2 (1.0)	15 (1.9)	0.569	0 (0.0)	2 (3.8)	0.999
Trauma	105 (6.0)	77 (5.6)	0.709	58 (9.9)	6 (4.7)	0.087
Smoke inhalation	34 (2.1)	22 (1.6)	0.412	0 (0.0)	1 (0.8)	0.999

eTable 5 - Baseline characteristics and clinical outcomes according to the missing data in tidal volume per predicted body weight

	No Missing			Missing		
	MIC (*n* = 1,762)	HIC (*n* = 1,379)	*p* value	MIC (*n* = 583)	HIC (*n* = 128)	*p* value
Pulmonary contusion	29 (1.8)	51 (3.7)	0.001	0 (0.0)	1 (0.9)	0.999
Burn	2 (1.0)	4 (0.5)	0.769	---	---	---
Pulmonary vasculitis	1 (0.5)	7 (0.9)	0.918	---	---	
Non–cardiogenic shock	278 (15.8)	118 (8.6)	< 0.001	41 (7.0)	11 (8.6)	0.669
Near–drowning	3 (0.2)	1 (0.1)	0.745	---	---	---
Drug overdose	4 (2.0)	22 (2.7)	0.710	0 (0.0)	1 (1.9)	0.999
TRALI	6 (3.0)	25 (3.1)	0.999	2 (16.7)	1 (1.9)	0.155
Limitation of support	57 (3.5)	184 (13.4)	< 0.001	8 (14.0)	19 (17.4)	0.733
Development of ARDS	107 (6.2)	144 (10.8)	< 0.001	77 (14.2)	7 (8.1)	0.172
Duration of ventilation, days	3 (1 - 6)	4 (2 - 10)	< 0.001	5 (2 - 9)	2 (1 - 5)	< 0.001
ICU length of stay, days	4 (2 - 9)	7 (3 - 14)	< 0.001	11 (6 - 18)	5 (3 - 11)	< 0.001
ICU mortality	494 (29.0)	263 (19.8)	< 0.001	190 (35.1)	20 (22.5)	0.026

Data are median (quartile 25% – quartile 75%) or N (%)

Data on LIPS available in 2250 (58.4%) of patients.

MIC, low– and middle–income countries; HIC, high–income countries; BMI: body mass index; LIPS: Lung Injury Prediction Score; ARDS: acute respiratory distress syndrome; SOFA: Sequential Organ Failure Assessment; COPD: chronic obstructive pulmonary disease; TRALI: transfusion–related acute lung injury

LIST OF INVESTIGATORS FROM INCLUDED STUDIES

ERICC study

INVESTIGATORS:

Luciano CP Azevedo, Marcelo Park (Research and Education Institute, Hospital Sírio-Libanês, São Paulo, Brazil; & ICU, Emergency Medicine Department, Hospital das Clinicas da Faculdade de Medicina da Universidade de São Paulo, São Paulo, Brazil); Jorge IF Salluh, Marcio Soares (D'Or Institute for Research and Education, Rio de Janeiro, Brazil); Alvaro Rea-Neto, Mirella C Oliveira (CEPETI - Centro de Estudos e Pesquisas em Terapia Intensiva, Curitiba, Brazil); Vicente C Souza-Dantas (ICU, Instituto Nacional de Câncer - Hospital do Câncer I, Rio de Janeiro, Brazil; ICU, Hospital Pasteur, Rio de Janeiro, Brazil); Pedro Varaschin (ICU, Hospital Pasteur, Rio de Janeiro, Brazil); Paulo Fernando GMM Tierno (ICU, Surgical Emergency Department, Hospital das Clinicas da Faculdade de Medicina da Universidade de São Paulo, São Paulo, Brazil); Felipe dal-Pizzol (ICU, Hospital São José, Criciúma, 88801-250, Brazil); Ulysses VA Silva (ICU, Fundação Pio XII, Hospital de Câncer de Barretos, Barretos, Brazil); Marcos Knivel (ICU, Hospital São Lucas, Rio de Janeiro, Brazil); Antonio P Nassar Jr (ICU, Hospital São Camilo Pompéia, São Paulo, Brazil); Rossine A Alves (ICU, Hospital Regional Público do Araguaia, Redenção, Brazil); Juliana C Ferreira (ICU, Hospital A. C. Camargo, São Paulo, Brazil); Cassiano Teixeira (ICU, Hospital Moinhos de Vento, Porto Alegre, Brazil); Valeria Rezende (ICU, Hospital Geral de Roraima, Boa Vista, Brazil); Amadeu Martinez (ICU, Hospital Espanhol, Salvador, Brazil); Paula M Luciano (ICU, Hospital Estadual Américo Brasiliense, Américo Brasiliense, Brazil); Guilherme Schettino (Research and Education Institute, Hospital Sírio-Libanês, São Paulo, Brazil)

SITE INVESTIGATORS:

BAHIA: Hospital Espanhol - Salvador (Amadeu Martinez, Lívia Leal, Antonio Jorge Pereira).

DISTRITO FEDERAL: Hospital Santa Luzia - Brasilia (Marcelo de Oliveira Maia, José Aires Neto).

Espírito Santo: Vitória Apart Hospital - Vitória (Claudio Piras), Centro Integrado de Atenção à Saude (CIAS) Unimed Vitória - Vitória (Eliana Bernadete Caser, Cora Lavigne Moreira), Hospital Meridional - Cariacica (Pablo Braga Gusman, Dyanne Moysés Dalcomune).

MARANHÃO: UDI Hospital - São Luís (Alexandre Guilherme Ribeiro de Carvalho, Louise Aline Romão Gondim, Lívia Mariane Castelo Branco Reis)

MINAS GERAIS: Hospital Madre Tereza - Belo Horizonte (Daniel da Cunha Ribeiro, Leonardo de Assis Simões, Rafaela Siqueira Campos, José Carlos Fernandez Versiani dos Anjos), Hospital Mater Dei - Belo Horizonte (Frederico Bruzzi Carvalho).

PARÁ: Hospital Regional Público do Araguaia - Redenção (Rossine Ambrosio Alves, Lilian Batista Nunes).

PARANÁ: Hospital do Trabalhador - Curitiba (Álvaro Réa-Neto, Mirella Cristine de Oliveira), Hospital Vita Batel - Curitiba (Álvaro Réa-Neto, Mirella Cristine de Oliveira), Hospital Universitario Cajuru - Curitiba (Álvaro Réa-Neto, Luana Tannous), Instituto de Neurologia de Curitiba (INC) - Curitiba (Álvaro Réa-Neto, Brenno Cardoso Gomes).

RIO DE JANEIRO: Instituto Nacional de Câncer - Hospital do Câncer I - Rio de Janeiro (Vicente Cés de Souza Dantas), Hospital de Clínicas de Niterói - Niterói (Fernando Borges Rodriguez, Priscila Abelha); Hospital de Clínicas Mario Lioni - Duque de Caxias (Marcelo E. Lugarinho); Instituto de Pesquisa Clínica Evandro Chagas (IPEC) - Rio de Janeiro (Andre Japiassu), Hospital da Mulher Heloneida Studart - Rio de Janeiro (Hélder Konrad de Melo, Elton Afonso Lopes), Hospital Pasteur - Rio de Janeiro (Pedro Varaschin, Vicente Cés de Souza Dantas), Hospital São Lucas - Rio de Janeiro (Marcos Freitas Knibel, Micheli Ponte, Pedro Mendes de Azambuja Rodrigues), Hospital Pro-Cardiaco - Rio de Janeiro (Rubens Carmo Costa Filho, Felipe Saddy, Théia Forny Wanderley Castellões, Suzana Alves Silva), Nortecor Hospital de Clínica - Rio de Janeiro (Luiz Antonio Gomes Osorio, Dora Mannarino), Hospital Copa D'Or - Rio de Janeiro (Rodolfo Espinoza, Cassia Righy, Marcio Soares, Jorge Salluh, Lilian Tanaka, Daniel Aragão, Maria Eduarda Tavares, Maura Goncalves Pereira Kehdi).

RORAIMA: Hospital Geral de Roraima - Boa Vista (Valéria Maria Campos Rezende, Roberto Carlos Cruz Carbonell).

RIO GRANDE DO SUL: Hospital Moinhos de Vento - Porto Alegre (Cassiano Teixeira, Roselaine Pinheiro de Oliveira, Juçara Gasparetto Maccari, Priscylla Souza Castro), Santa Casa de Misericórdia de Porto Alegre - Pavilhão Pereira Filho (Paula Berto, Patricia Schwarz); Santa Casa de Misericórdia de Porto Alegre - Hospital Santa Rita (André Peretti Torelly, Thiago Lisboa, Paula Berto, Edison Moraes).

SANTA CATARINA: Hospital São José - Criciúma (Felipe Dal-Pizzol, Cristiane Tomasi Damiani, Cristiane Ritter).
SÃO PAULO: Hospital A. C. Camargo - São Paulo (Juliana Carvalho Ferreira, Ramon Teixeira Costa, Pedro Caruso); Fundação Pio XII - Hospital de Câncer de Barretos - Barretos (Cristina Prata Amendola, Amanda Maria R. R. de Oliveira, Ulysses V. A. Silva, Luciana Coelho Sanches, Rosana D. S. Almeida); Hospital Sírio Libanês - São Paulo (Luciano Cesar Azevedo, Marcelo Park, Guilherme Schettino), Hospital Israelita Albert Einstein - São Paulo (Murillo Santucci Assunção, Eliezer Silva), Hospital São Camilo Santana - São Paulo (Carlos Eduardo Barboza, Antonio Paulo Nassar Junior), Hospital São Camilo Pompéia - São Paulo (Antonio Paulo Nassar Junior), Hospital das Clinicas da Faculdade de Medicina da USP - UTI Disciplina Emergências Clinicas - São Paulo (Luciano Cesar Azevedo, Marcelo Park), Hospital das Clinicas da Faculdade de Medicina da USP - UTI Disciplina Emergências Cirurgicas - São Paulo (Paulo Fernando Guimarães Morando Marzocchi Tierno, Luis Marcelo Malbouisson, Lucas Oliveira), Hospital das Clinicas da Faculdade de Medicina da USP - UTI Disciplina Anestesiologia - São Paulo (Davi Cristovao), Hospital Ipiranga - Rede Amil - São Paulo (Manoel Leitão Neto, Ênio Rego, Fernanda Eugênia Fernandes), Hospital Do Coração - São Paulo (Marcelo Luz Pereira Romano, Alexandre Biasi Cavalcanti, Dalton de Souza Barros, Érica Aranha Suzumura, Karla Loureiro Meira, Gustavo Affonso de Oliveira), Hospital Estadual de Américo Brasiliense - Américo Brasiliense (Paula Menezes Luciano, Evelin Drociunas Pacheco), Hospital São Paulo da Universidade Federal de São Paulo - São Paulo (Bruno Franco Mazza, Flavia Ribeiro Machado, Elaine Ferreira), Hospital Universitário da Universidade de São Paulo - São Paulo (Ronaldo Batista dos Santos, Alexandra Siqueira Colombo, Antonio Carlos Nogueira, Juliana Baroni Fernandes, Raquel Siqueira Nóbrega, Barbara do C.S. Martins, Francisco Soriano), Hospital São Luiz Jardim Anália Franco - São Paulo (Rafaela Deczka Morsch, Andre Luiz Baptiston Nunes), Instituto do Câncer do Estado de São Paulo (ICESP) - São Paulo (Juliano Pinheiro de Almeida, Ludhmila Hajjar, Sílvia Moulin), Hospital e Maternidade São Luiz - Unidade Itaim - São Paulo (Fábio Poianas Giannini, Andre Luiz Baptiston Nunes).

LUNG SAFE study

NATIONAL COORDINATORS:
Argentina: Fernando Rios; Australia/New Zealand: Frank Van Haren; Bangladesh: Mohammad Omar Faruq; Belgium: Sottiaux T, Depuydt P; Bolivia: Fredy S Lora; Brazil: Luciano Cesar Azevedo; Canada: Eddy Fan; Chile: Guillermo Bugedo ; China: Haibo Qiu; Colombia: Marcos Gonzalez; Costa Rica: Juan Silesky; Czech Republic: Vladimir Cerny; Denmark: Jonas Nielsen; Ecuador: Manuel Jibaja; France: Tài Pham; Germany: Hermann Wrigge; Greece: Dimitrios Matamis; Guatemala: Jorge Luis Ranero; Hong Kong: Charles Gomersall; India: Pravin Amin; Iran: S.M. Hashemian; Ireland: Kevin Clarkson; Italy: Giacomo Bellani; Japan: Kiyoyasu Kurahashi; Korea: Younsuck Koh; Mexico: Asisclo Villagomez; Morocco: Amine Ali Zeggwagh; Netherlands: Leo M Heunks; Norway: Jon Henrik Laake ; Pakistan: Waqar Kashif ; Panama: Jorge Synclair; Philippines: Jose Emmanuel Palo ; Portugal: Antero do Vale Fernandes; Romania: Dorel Sandesc; Saudi Arabia: Yaasen Arabi; Serbia: Vesna Bumbasierevic; Spain: Nicolas Nin, Jose A Lorente; Sweden: Anders Larsson; Switzerland: Lise Piquilloud; Thailand: Boonsong Patjanasoontorn ; Tunisia: Fekri Abroug; United Kingdom: Daniel F McAuley, Lia McNamee; Uruguay: Javier Hurtado; USA: Ed Bajwa; Venezuela: Gabriel Démpaire;
NATIONAL SOCIETIES/NETWORKS ENDORSING THE STUDY:
ANZICS Clinical Trials Group, Réseau Européen de Recherche en Ventilation Artificielle (ReVA Network); Irish Critical Care Trials Group; Société de Réanimation de Langue Française (SRLF); Société Française d’Anesthésie et de Réanimation (SFAR); Società Italiana Anestesia, Analgesia, Rianimazione e Terapia Intensiva (SIAARTI); The Japanese Society of Intensive Care Medicine (JSICM); Nonprofit Organization Japanese Society of Education for Physicians and Trainees in Intensive Care (JSEPTIC); UK Intensive Care Society.
STUDY COORDINATION: Guy M Francois (European Society of Intensive Care Medicine, Brussels, Belgium)
DATA REVISION AND MANAGEMENT: Francesca Rabboni (University Of Milan- Bicocca, Monza, Italy), Fabiana Madotto (University Of Milan-Bicocca, Monza, Italy), Sara Conti (University Of Milan-Bicocca, Monza, Italy)
SITE INVESTIGATORS BY COUNTRY:
ALBANIA: Uhc Mother Theresa (Tirana): Hektor Sula, Lordian Nunci; University Hospital Shefqet Ndroqi (Tirana): Alma Cani;
ARGENTINA: Clinica De Especialidades (Villa Maria): Alan Zazu ; Hospital Dr Julio C. Perrando (Resistencia): Christian Dellera, Carolina S Insaurralde; Sanatorio Las Lomas (San Isidro, Buenos Aires): Risso V Alejandro; Sanatorio De La Trinidad San Isidro (San Isidro): Julio Daldin, Mauricio

Vinzio; Hospital Español De Mendoza (Godoy Cruz - Mendoza): Ruben O Fernandez; Hospital Del Centenario (Rosario): Luis P Cardonnet, Lisandro R Bettini; San Antonio (Gualeguay (Entre Rios)): Mariano Carboni Bisso, Emilio M Osman; Cemic (Buenos Aires): Mariano G Setten, Pablo Lovazzano; Hospital Universitrario Austral (Pilar): Javier Alvarez, Veronica Villar; Hospital Por + Salud (Pami) Dr. Cesar Milstein (Buenos Aires): Norberto C Pozo, Nicolas Grubissich; Sanatorio Anchorena (Buenos Aires): Gustavo A Plotnikow, Daniela N Vasquez; Sanatorio De La Trinidad Mitre (Buenos Aires): Santiago Ilutovich, Norberto Tiribelli; Hospital Luis Lagomaggiore (Mendoza): Ariel Chena, Carlos A Pellegrini; H.I.G.A San Martín (La Plata): María G Saenz, Elisa Estenssoro; Hospital Misericordia (Cordoba): Matias Brizuela, Hernan Gianinetto; Sanatorio Juncal (Temperley): Pablo E Gomez, Valeria I Cerrato; Hospital D. F. Santojanni (Buenos Aires): Marco G Bezzi, Silvina A Borello; Hospital Alejandro Posadas (Buenos Aires): Flavia A Loiacono, Adriana M Fernandez;
AUSTRALIA: St. Vincents Hospital, Sydney (Darlinghurst): Serena Knowles, Claire Reynolds; St George Public Hospital (Kogarah): Deborah M Inskip, Jennene J Miller; Westmead Hospital (Westmead): Jing Kong, Christina Whitehead; Flinders Medical Centre (Bedford Park, South Australia): Shailesh Bihari; John Hunter Hospital (Newcastle): Aylin Seven, Amanda Krstevski; Canberra Hospital (Garran): Helen J Rodgers, Rebecca T Millar; Calvary Mater Newcastle (Waratah): Toni E Mckenna, Irene M Bailey; Cabrini Hospital (Melbourne): Gabrielle C Hanlon; Liverpool Hospital (Liverpool): Anders Aneman, Joan M Lynch; Coffs Harbour Health Campus (Coffs Harbour): Raman Azad, John Neal; Sir Charles Gairdner Hospital (Nedlands): Paul W Woods, Brigit L Roberts; Concord Hospital (Concord): Mark R Kol, Helen S Wong;
AUSTRIA: General Hospital Of Vienna/Medical University Of Vienna (Vienna): Katharina C Riss, Thomas Staudinger;
BELGIUM: Cliniques universitaires St Luc, UCL (Brussels): Xavier Wittebole, Caroline Berghe; CHU Dinant-Godinne (Yvoir): Pierre A Bulpa, Alain M Dive; AZ Sint Augustinus Veurne (Veurne): Rik Verstraete, Herve Lebbinck; Ghent University Hospital (Ghent): Pieter Depuydt, Joris Vermassen;; University Hospitals Leuven (Leuven): Philippe, Meersseman, Helga Ceunen;
BRAZIL: Hospital Renascentista (Pouso Alegre): Jonas I Rosa, Daniel O Beraldo; Vitoria Apart Hospital (Serra): Claudio Piras, Adenilton M Rampinelli; Hospital Das Clinicas (São Paulo): Antonio P Nassar Jr; Hospital Geral Do Grajaù (São Paulo): Sergio Mataloun, Marcelo Moock; Evangelical Hospital (Cachoeiro De Itapemirim / Espírito Santo): Marlus M Thompson, Claudio H Gonçalves-,; Hospital Moinhos De Vento (Porto Alegre): Ana Carolina P Ant ônio, Aline Ascoli; Hospital Alvorada Taguatinga (Taguatinga): Rodrigo S Biondi, Danielle C Fontenele; Complexo Hospitalar Mngabeira Tarcisio Burity (Joao Pessoa): Danielle Nobrega, Vanessa M Sales;
BRUNEI DARUSSALAM: Raja Isteri Pengiran Anak Saleha (Ripas) Hospital (Bandar Seri Begawan): Dr Suresh .Shindhe, Dr Dk Maizatul Aiman B Pg Hj Ismail;
CANADA: Medical-Surgical ICU of St Michael's Hospital (Toronto): John Laffey, Francois Beloncle; St. Josephs Health Centre (Toronto): Kyle G Davies, Rob Cirone; Sunnybrook Health Sciences Center (Toronto): Venika Manoharan, Mehvish Ismail; Toronto Western Hospital (Toronto): Ewan C Goligher, Mandeep Jassal; Medical Surgical ICU of the Toronto General Hospital (Toronto): Niall D. Ferguson, Erin Nishikawa, Areej Javeed; Cardiovascular ICU of St Michael's Hospital (Toronto): Gerard Curley, Nuttapol Rittayamai ; Cardiovascular ICU of the Toronto General Hospital (Toronto): Matteo Parotto, Mount Sinai Hospital (Toronto): Sangeeta Mehta, Jenny Knoll ; Trauma-Neuro ICU of St Michael's Hospital (Toronto): Antoine Pronovost, Sergio Canestrini
CHILE: Hospital Clínico Pontificia Universidad Católica De Chile (Santiago): Alejandro R Bruhn, Patricio H Garcia; Hospital Militar De Santiago (Santiago): Felipe A Aliaga, Pamela A Farías; Clinica Davila (Santiago): Jacob S Yumha; Hospital Guillermo Grant Benavente (Concepcion): Claudia A Ortiz, Javier E Salas; Clinica Las Lilas (Santiago): Alejandro A Saez, Luis D Vega; Hospital Naval Almirante Nef (Viña Del Mar): Eduardo F Labarca, Felipe T Martinez; Hospital Luis Tisné Brousse (Penanolen): Nicolás G Carreño, Pilar Lora;
CHINA: The Second Affiliated Hospital Of Harbin Medical University (Harbin): Haitao Liu; Nanjing Zhong-Da Hospital, Southeast University (Nanjing): Haibo Qiu, Ling Liu; The First Affiliated Hospital Of Anhui Medical University (Hefei): Rui / Tang, Xiaoming Luo; Peking University People's Hospital (Beijing): Youzhong An, Huiying Zhao; Fourth Affiliated Hospital Of Harbin Medical University (Harbin): Yan - Gao, Zhe - Zhai; Nanjing Jiangbei Peoples Hospital Affiliated To Medical School Of Southeast University (Nanjing): Zheng L Ye, Wei Wang; The First Affiliated Hospital Of Dalian Medical Unvercity (Dalian): Wenwen Li, Qingdong Li; Subei Peoples Hospital Of Jiangsu Province (Yanghzou): Ruiqiang Zheng ; Jinling Hospital (Nanjing): Wenkui Yu, Juanhong Shen; Urumqi General Hospital (Urumqi): Xinyu Li; Intensive Care Unit, First Affiliated Hospital Of Wanna Medical College, Yijishan Hospital, (Wuhu): Tao Yu, Weihua Lu; Sichuan Provincial Peoples Hospital (Chengdu): Ya Q Wu, Xiao B Huang; Hainan Province Peoples Hospital (Haikou): Zhenyang He; Peoples Hospital Of Jiangxi

Province (Nanchang): Yuanhua Lu; Qilu Hospital Of Shandong University (Jinan): Hui Han, Fan Zhang; Zhejiang Provincial Peoples Hospital (Hangzhou): Renhua Sun ; The First Affiliated Hospital Of Bengbu Medical College (Bengbu, Anhui): Hua X Wang, Shu H Qin; Nanjing Municipal Government Hospital (Nanjing): Bao H Zhu, Jun Zhao; The First Hospital Of Lanzhou University (Lanzhou): Jian / Liu, Bin / Li; The First Affiliated Hospital Of Chongqing University Of Medical Science (Chongqing): Jing L Liu, Fa C Zhou; Xuzhou Central Hospital, Jiangsu Province, China (Xuzhou): Qiong J Li, Xing Y Zhang; The First Peoples Hospital Of Foshan (Foshan): Zhou Li-Xin, Qiang Xin-Hua; The First Affiliated Hospital Of Guangxi Medical University (Nanning): Liangyan Jiang; Renji Hospital ,Shanghai Jiao Tong University School Of Medicine (Shanghai): Yuan N Gao, Xian Y Zhao; First Hospital Of Shanxi Medical University (Taiyuan): Yuan Y Li, Xiao L Li; Shandong Provincial Hospital (Jinan): Chunting Wang, Qingchun Yao ; Fujian Provincial Hospital (Fuzhou): Rongguo Yu, Kai Chen;
Henan Provincial People’s Hospital (Zhengzhou): Huanzhang Shao, Bingyu Qin ; The Second Affiliated Hospital Of Kunming Medical University (Kunming City): Qing Q Huang, Wei H Zhu; Xiangya Hospital, Central South University (Changsha): Ai Y Hang, Ma X Hua; The First Affiliated Hospital Of Guangzhou Medical University (Guangzhou): Yimin Li, Yonghao Xu; Peoples Hospital of Hebei Province (Shijiazhuang): Yu D Di, Long L Ling; Guangdong General Hospital (Guangzhou): Tie H Qin, Shou H Wang; Beijing Tongren Hospital (Beijing): Junping Qin; Jiangsu Province Hospital (Nanjing): Yi Han, Suming Zhou;COLOMBIA: Fundación Valle Del Lili (Cali): Monica P Vargas;
COSTA RICA: Hospital San Juan De Dios (): Juan I Silesky Jimenez, Manuel A González Rojas; Hospital San Juan De Dios (San José): Jaime E Solis-Quesada, Christian M Ramirez- Alfaro;
CZECH REPUBLIC: University Hospital Of Ostrava (Ostrava): Jan Máca, Peter Sklienka; DENMARK: Aarhus Universitetshospital (Aarhus N): Jakob Gjedsted, Aage Christiansen;
Rigshopitalet: Jonas Nielsen;
ECUADOR: Hospital Militar (Quito): Boris G Villamagua, Miguel Llano;
FRANCE: Clinique du Millénaire (Montpellier): Philippe Burtin, Gautier Buzancais; Centre Hospitalier (Roanne): Pascal Beuret, Nicolas Pelletier; CHU d’Angers (Angers): Satar Mortaza, Alain Mercat; Hôpital Marc Jacquet (Melun): Jonathan Chelly, Sébastien Jochmans; CHU de Caen (Caen): Nicolas Terzi, Cédric Daubin; Henri Mondor Hospital (Créteil): Guillaume Carteaux, Nicolas de Prost; Cochin Hospital (Paris): Jean-Daniel Chiche, Fabrice Daviaud ; CHU Tenon (Paris): Tài Pham, Muriel Fartoukh; CH Mulhouse-Emile Muller (Mulhouse): Guillaume Barberet, Jerome Biehler; Archet 1 University Hospital (Nice): Jean Dellamonica, Denis Doyen; Hopital Sainte Musse (Toulon): Jean-Michel Arnal, Anais Briquet; Hopital Nord - Réanimation des Détresses Respiratoires et Infections Sévères (Marseille): Fanny Klasen, Laurent Papazian; HEGP (Paris): Arnaud Follin; Louis Mourier Hospital (Colombes): Damien Roux, Jonathan Messika; Centre Hospitalier de Dax (Dax): Evangelos Kalaitzis ; Réanimation Médicale, GH Pitié-Salpêtrière (Paris): Laurence Dangers, Alain Combes; Ap-Hp Ambroise Paré (Boulogne-Billancourt): Siu-Ming Au; University Hospital Rouen (Rouen): Gaetan Béduneau, Dorothée Carpentier; CHU Amiens (Amiens - Salouel): Elie H Zogheib, Herve Dupont; Centre Hospitalier Intercommunal Robert Ballanger (Aulnay Sous Bois): Sylvie Ricome, Francesco L Santoli; Centre Hospitalier René Dubos (Pontoise): Sebastien L Besset; CHI Portes de l’Oise (Beaumont Sur Oise): Philippe Michel, Bruno Gelée; Archet 2 University Hospital (Nice): Pierre-Eric Danin, Bernard Goubaux; Centre Hospitalier Pierre Oudot (Bourgoin Jallieu): Philippe J Crova, Nga T Phan; CH Dunkerque (Dunkerque): Frantz Berkelmans; Centre Hospitalier de Belfort Montbéliard (Belfort): Julio C Badie, Romain Tapponnier; Centre Hospitalier Emile Muller (Mulhouse): Josette Gally, Samy Khebbeb; Hôpital de Hautepierre- Hôpitaux Universitaires de Strasbourg (Strasbourg): Jean-Etienne Herbrecht, Francis Schneider; Centre Hospitalier de Dieppe (Dieppe): Pierre-Louis M Declercq, Jean-Philippe Rigaud; Bicêtre (Le Kremin-Bicetre): Jacques Duranteau, Anatole Harrois; CHU Gabriel Montpied (Clermont- Ferrand): Russell Chabanne, Julien Marin; CHU Estaing (Clermont-Ferrand): Jean-Michel Constantin, Sandrine Thibault; CHI Eure-Seine Evreux (Evreux): Mohammed Ghazi, Messabi Boukhazna; Centre Hospitalier de Châlons en Champagne (Châlons en Champagne): Salem Ould Zein; CH Beauvais (Beauvais): Jack R Richecoeur, Daniele M Combaux; Centre Hospitalier Le Mans (Le Mans): Fabien Grelon, Charlene Le Moal; Hôpital Fleyriat (Bourg en Bresse): Elise P Sauvadet, Adrien Robine; Hôpital Saint Louis (Paris): Virginie Lemiale, Danielle Reuter; Pneumologie et Réanimation Médicale, Hôpital Pitié-Salpêtrière (Paris): Martin Dres, Alexandre Demoule; Centre Hospitalier Gonesse (Gonesse): Dany Goldgran-Toledano; Hôpital Croix Rousse (Lyon): Loredana Baboi, Claude Guérin;
GERMANY: St. Nikolaus-Stiftshospital (Andernach): Ralph Lohner; Fachkrankenhaus Coswig Gmbh (Coswig):Jens Kraßler, Susanne Schäfer; University Hospital Frankfurt (Frankfurt am Main): Kai D Zacharowski, Patrick Meybohm; Department of Anaesthesia & Intensive Care Medicine, University Hospital of Leipzig (Leipzig): Andreas W Reske, Philipp Simon; Asklepios Klinik Langen (Langen): Hans-Bernd F Hopf, Michael Schuetz; Städtisches Krankenhaus Heinsberg (Heinsberg): Thomas

Baltus;
GREECE: Hippokrateion General Hospital Of Athens (Athens): Metaxia N Papanikolaou, Theonymfi G Papavasilopoulou; Gh Ahepa (Thessaloniki): Giannis A Zacharas, Vasilis Ourailogloy; Hippokration General Hospital of Thessaloniki (Thessaloniki): Eleni K Mouloudi, Eleni V Massa; Hospital General of Kavala (Kavala): Eva O Nagy, Electra E Stamou; Papageorgiou General Hospital (Thessaloniki): Ellada V Kiourtzieva, Marina A Oikonomou;
GUATEMALA: Hospital General De Enfermedades, Instituto Guatemalteco De Seguridad Social (Ciudad De Guatemala): Luis E Avila; Centro Médico Militar (Guatemala): Cesar A Cortez, Johanna E Citalán;
INDIA: Deenanath Mangeshkar Hospital And Research Center (Pune): Sameer A Jog, Safal D Sable; Care Institute Of Medical Sciences (CIMS) Hospital (Ahmedabad): Bhagyesh Shah ; Sanjay Gandhi Postgraduate Institute Of Medical Sciences (SGPGIMS) (Lucknow): Mohan Gurjar, Arvind K Baronia; Rajasthan Hospital (Ahmedabad): Mohammedfaruk Memon ; National Institute Of Mental Health And Neuro Sciences (NIMHANS) (Bangalore): Radhakrishnan Muthuchellappan, Venkatapura J Ramesh; Anaesthesiology Unit of the Kasturba Medical College & Dept of Respiratory Therapy, SHOAS, Manipal University (Manipal): Anitha Shenoy, Ramesh Unnikrishnan; Sanjeevan Hospital (Pune): Subhal B Dixit, Rachana V Rhayakar; Apollo Hospitals (Chennai): Nagarajan Ramakrishnan ,Vallish K Bhardwaj; Medicine Unit of the Kasturba Medical College & Dept of Respiratory Therapy, SHOAS, Manipal University (Manipal): Heera L Mahto, Sudha V Sagar; G Kuppuswamy Naidu Memorial Hospital (Coimbatore): Vijayanand Palaniswamy, Deeban Ganesan;
IRAN: NRITLD/Masih Daneshvari (Tehran): Seyed Mohammadreza Hashemian, Hamidreza Jamaati ; Milad Hospital (Tehran): Farshad Heidari
IRELAND: St Vincent’s University Hospital (Dublin): Edel A Meaney, Alistair Nichol; Mercy University Hospital (Cork): Karl M Knapman, Donall O’Croinin ; Cork University Hospital (Cork): Eimhin S Dunne, Dorothy M Breen; Galway University Hospital (Galway): Kevin P Clarkson, Rola F Jaafar; Beaumont Hospital (Dublin): Rory Dwyer, Fahd Amir; Mater Misericordiae University Hospital (Dublin): Olaitan O Ajetunmobi, Aogan C O’Muircheartaigh; Tallaght Hospital (Dublin): Colin S Black, Nuala Treanor; Saint James’s Hospital (Dublin): Daniel V Collins, Wahid Altaf;
ITALY: Santa Maria delle Croci Hospital (Ravenna): Gianluca Zani, Maurizio Fusari; Arcispedale Sant’Anna Ferrara. (Ferrara): Savino Spadaro, Carlo A Volta; Ospedale Profili (Fabriano) (An): Romano Graziani, Barbara Brunettini; Umberto I Nocera Inferiore (Nocera Inferiore Salerno): Salvatore Palmese; Azienda Ospedaliera San Paolo – Polo Universitario- Università degli Studi di Milano (Milan): Paolo Formenti, Michele Umbrello; Sant’Anna (San Fermo Della Battaglia (Co)): Andrea Lombardo; Spedali Civili Brescia (Brescia): Elisabetta Pecci, Marco Botteri; Fondazione Irccs Ca Granda, Ospedale Maggiore Policlinico (Milan): Monica Savioli, Alessandro Protti; University Campus Bio-Medico of Rome (Rome): Alessia Mattei, Lorenzo Schiavoni; Azienda Ospedaliera "Mellino Mellini" (Chiari (Bs)): Andrea Tinnirello, Manuel Todeschini; Policlinico P. Giaccone, University of Palermo (Palermo): Antonino Giarratano, Andrea Cortegiani; Niguarda Cà Granda Hospital (Milan): Sara Sher, Anna Rossi; A.Gemelli University Hospital (Rome): Massimo M Antonelli, Luca M Montini; Ospedale "Sandro Pertini" (Rome): Paolo Casalena, Sergio Scafetti; ISMeTT IRCCS UPMC (Palermo): Giovanna Panarello, Giovanna Occhipinti; Ospedale San Gerardo (Monza): Nicolò Patroniti, Matteo Pozzi; Santa Maria Della Scaletta (Imola): Roberto R Biscione, Michela M Poli; Humanitas Research Hospital (Rozzano): Ferdinando Raimondi, Daniela Albiero; Ospedale Desio - Ao Desio-Vimercate (Desio): Giulia Crapelli, Eduardo Beck; Pinetagrande Private Hospital (Castelvolturno): Vincenzo Pota, Vincenzo Schiavone; Irccs San Martino Ist (Genova): Alexandre Molin, Fabio Tarantino; Ospedale San Raffaele (Milano): Giacomo Monti, Elena Frati; Ospedali Riuniti Di Foggia (Foggia): Lucia Mirabella, Gilda Cinnella; Azienda Ospedaliera Luigi Sacco - Polo Universitario (Milano): Tommaso Fossali, Riccardo Colombo; A.O.U. Città della Salute e della Scienza di Torino (Turin): Pierpaolo Terragni Ilaria Pattarino; Università degli Studi di Pavia-Fondazione IRCCS Policlinico San Matteo (Pavia): Francesco Mojoli, Antonio Braschi; Ao Ospedale Civile Legnano (Legnano): Erika E Borotto; Arnas Ospedale Civico Di Cristina Benfratelli (Palermo): Andrea N Cracchiolo, Daniela M Palma; Azienda Ospedaliera Della Provincia Di Lecco - Ospedale "A. Manzoni" (Lecco): Francesco Raponi, Giuseppe Foti; A.O. Provincia Di Lecco - Ospedale Alessandro Manzoni (Lecco): Ettore R Vascotto, Andrea Coppadoro; Cliniche Universitarie Sassari (Sassari): Luca Brazzi, Leda Floris; IRCCS Policlinico San Matteo (Pavia): Giorgio A Iotti, Aaron Venti;
JAPAN: Yokohama City University Hospital (Yokohama): Osamu Yamaguchi, Shunsuke Takagi; Toyooka Hospital (Toyooka City,Hyogo Prefecture): Hiroki N Maeyama; Chiba University Hospital (Chiba City): Eizo Watanabe, Yoshihiro Yamaji; Okayma University Hospital (Okayama): Kazuyoshi Shimizu, Kyoko Shiozaki; Japanese Foundation for Cancer Research, Cancer Institute Hospital, Department Of Emergency Medicine And Critical Care (Tokyo): Satoru Futami; Ibaraki Prefectural

Central Hospital (Kasama): Sekine Ryosuke; Tohoku University Hospital (Sendai-Shi): Koji Saito, Yoshinobu Kameyama; Tokyo Medical University Hachioji Medical Center (Hachioji, Tokyo): Keiko Ueno; Tokushima University Hospital (Tokushima): Masayo . Izawa, Nao Okuda; Maebashi Red Cross Hospital (Gunma Maebashi): Hiroyuki Suzuki, Tomofumi Harasawa; Urasoe General Hospital (Urasoe): Michitaka Nasu, Tadaaki Takada; Ohta General Hospital Foundation Ohta Nishinouchi Hospital (Fukushima): Fumihito Ito; Jichi Medical University Hospital (Shimotsuke): Shin - Nunomiya, Kansuke - Koyama; Mito Kyodo General Hospital, Tsukuba University Hospital Mito Medical Center (Mito): Toshikazu Abe; Sendai City Hospital (Sendai): Kohkichi Andoh, Kohei Kusumoto; Ja Hiroshima General Hospital (Hatsukaichi City, Hiroshima): Akira Hirata, Akihiro Takaba; Yokohama Rosai Hospital (Yokohama): Hiroyasu Kimura; Nagasaki University Hospital (Nagasaki): Shuhei Matsumoto, Ushio Higashijima; Niigata University Medical & Dental Hospital (Niigata): Hiroyuki Honda, Nobumasa Aoki; Mie University Hospital (Tsu, Mie): Hiroshi Imai; Yamaguchi University Hospital (Ube, Yamaguchi): Yasuaki Ogino, Ichiko Mizuguchi; Saiseikai Kumamoto Hospital (Kumamoto City): Kazuya Ichikado; Shinshu University School Of Medecine (Matsumoto City): Kenichi Nitta, Katsunori Mochizuki; Kuki General Hospital (Kuki): Tomoaki Hashida; Kyoto Medical Center (Kyoto): Hiroyuki Tanaka ; Fujita Health University (Toyoake): Tomoyuki Nakamura, Daisuke Niimi; Rakwakai Marutamachi Hospital (Kyoto): Takeshi Ueda; Osaka University Hospital (Suita City, Osaka Prefecture): Yozo Kashiwa, Akinori Uchiyama;
LATVIA: Paul Stradins Clinical University Hospital (Riga): Olegs Sabelnikovs, Peteris Oss ; LEBANON: Kortbawi Hospital (Jounieh): Youssef Haddad ; MALAYSIA: Hospital Kapit (Kapit): Kong Y Liew;
MEXICO: Instituto Nacional De Cancerología, México (Mexico City): Silvio A Ñamendys- Silva, Yves D Jarquin-Badiola; Hospital De Especialidades "Antonio Fraga Mouret" Centro Medico Nacional La Raza IMSS (Mexico City): Luis A Sanchez-Hurtado, Saira S Gomez- Flores; Hospital Regional 1° De Octubre (Mexico City): Maria C Marin, Asisclo J Villagomez; Hospital General Dr Manuel Gea Gonzalez (Mexico City): Jordana S Lemus, Jonathan M Fierro; Hospital General De Zona No. 1 Instituto Mexicano Del Seguro Social Tepic Nayarit (Tepic): Mavy Ramirez Cervantes, Francisco Javier Flores Mejia; Centro Medico Dalinde (Mexico D.F.): Dulce Dector, Dulce M Dector; Opd Hospital Civil De Guadalajara Hospital Juan I Menchaca (Guadalajara): Daniel R Gonzalez, Claudia R Estrella; Hospital Regional De Ciudad Madero Pemex (Ciudad Madero): Jorge R Sanchez-Medina, Alvaro Ramirez-Gutierrez; Centro Médico ABC (Mexico D.F.): Fernando G George, Janet S Aguirre; Hospital Juarez De Mexico (Mexico City): Juan A Buensuseso, Manuel Poblano;
MOROCCO: Mohammed V University, University Teaching Ibn Sina Hospital (Rabat): Tarek Dendane, Amine Ali Zeggwagh; Hopital Militaire D'Instruction Mohammed V (Rabat): Hicham Balkhi; Errazi (Marrakech): Mina Elkhayari, Nacer Samkaoui; University Teaching Hospital Ibn Rushd (Casablanca): Hanane Ezzouine, Abdellatif Benslama; Hôpital des Spécialités de Rabat (HSR) (Rabat): Mourad Amor, Wajdi Maazouzi;
NETHERLANDS: Tjongerschans (Heerenveen): Nedim Cimic, Oliver Beck; Cwz (Nijmegen): Monique M Bruns, Jeroen A Schouten; Rijnstate Hospital (Arnhem): Myra - Rinia, Monique Raaijmakers; Radboud Umc (Nijmegen): Leo M Heunks, Hellen M Van Wezel; Maastricht University Medical Centre (Maastricht): Serge J Heines, Ulrich Strauch; Catharinaziekenhuis (Eindhoven): Marc P Buise; Academic Medical Center (Amsterdam): Fabienne D Simonis, Marcus J Schultz;
NEW ZEALAND: Tauranga Hospital (Tauranga): Jennifer C Goodson, Troy S Browne; Wellington Hospital (Wellington): Leanlove Navarra, Anna Hunt; Dunedin Hospital (Dunedin): Robyn A Hutchison, Mathew B Bailey; Auckland City Hospital (Auckland): Lynette Newby, Colin Mcarthur; Whangarei Base Hospital (Whangarei): Michael Kalkoff, Alex Mcleod; North Shore Hospital (Auckland): Jonathan Casement, Danielle J Hacking;
NORWAY: Ålesund Hospital (Ålesund): Finn H Andersen, Merete S Dolva; Oslo University Hospital - Rikshospitalet Medical Centre (Oslo): Jon H Laake, Andreas Barratt-Due; Stavanger University Hospital (Stavanger): Kim Andre L Noremark, Eldar Søreide; Haukeland University Hospital (Bergen): Brit Å Sjøbø, Anne B Guttormsen;
PERU: Hospital Nacional Edgardo Rebagliati Martins (Lima): Hector H Leon Yoshido; Clínica Ricardo Palma (Lima): Ronald Zumaran Aguilar, Fredy A Montes Oscanoa;
PHILIPPINES: The Medical City (Pasig): Alain U Alisasis, Joanne B Robles; Chong Hua Hospital (Cebu): Rossini Abbie B Pasanting-Lim, Beatriz C Tan;
POLAND: Warsaw University Hospital (Warsaw): Pawel Andruszkiewicz, Karina Jakubowska;
PORTUGAL: Centro Hospitalar Da Cova Da Beira (Covilhã): Cristina M Coxo; Hospital Santa Maria, Chln (Lisboa): António M Alvarez, Bruno S Oliveira; Centro Hospitalar Trás-Os-Montes E Alto Douro - Hospital De S.Pedro -Vila Real (Vila Real): Gustavo M Montanha, Nelson C Barros; Hospital Beatriz Ângelo (Loures): Carlos S Pereira, António M Messias; Hospital De Santa Maria (Lisboa): Jorge M

Monteiro; Centro Hospitalar Médio Tejo - Hospital De Abrantes (Abrantes): Ana M Araujo, Nuno T Catorze; Instituto Português De Oncologia De Lisboa (Lisboa): Susan M Marum, Maria J Bouw; Hospital Garcia De Orta (Almada): Rui M Gomes, Vania A Brito; Centro Hospitalar Do Algarve (Faro): Silvia Castro, Joana M Estilita; Hpp Hospital De Cascais (Alcabideche): Filipa M Barros; Hospital Prof. Doutor Fernando Fonseca Epe (Amadora): Isabel M Serra, Aurelia M Martinho;

ROMANIA: Fundeni Clinical Institute (Bucharest): Dana R Tomescu, Alexandra Marcu; Emergency Clinical County Hospital Timisoara (Timisoara): Ovidiu H Bedreag, Marius Papurica; Elias University Emergency Hospital (Bucharest): Dan E Corneci, Silvius Ioan Negoita;

RUSSIAN FEDERATION: University Hospital (Kemerovo): Evgeny Grigoriev; Krasnoyarsk Regional Hospital, Krasnoyarsk State Medical University (Krasnoyarsk): Alexey I Gritsan, Andrey A Gazenkampf;

SAUDI ARABIA: GICU of PSMMC (Riyadh): *Ghaleb Almekhlafi, Mohamad M Albarrak*; SICU of PSMMC (Riyadh): Ghanem M Mustafa;; King Faisal Hospital And Research Center (Riyadh): Khalid A Maghrabi, Nawal Salahuddin; King Fahad Hospital (Baha): Tharwat M Aisa; King Abdulaziz Medical City (Riyadh): Ahmed S Al Jabbary, Edgardo Tabhan; King Abdulaziz Medical City (Riyadh): Yaseen M Arabi; King Abdulaziz Medical City (Riyadh): Yaseen M Arabi, Olivia A Trinidad; King Abdulaziz Medical City (Riyadh): Hasan M Al Dorzi, Edgardo E Tabhan;

SOUTH AFRICA: Charlotte Maxeke Johannesburg Academic Hospital (Johannesburg): Stefan Bolon, Oliver Smith;

SPAIN: Hospital Sant Pau (Barcelona): Jordi Mancebo, Hernan Aguirre-Bermeo; Hospital Universitari Bellvitge (L Hospitalet De Llobregat (Barcelona)): Juan C Lopez-Delgado, Francisco Esteve; Hospital Son Llatzer (Palma De Mallorca): Gemma Rialp, Catalina Forteza; Sabadell Hospital, CIBER Enfermedades Respiratorias (Sabadell): Candelaria De Haro, Antonio Artigas; Hospital Universitario Central De Asturias (Oviedo): Guillermo M Albaiceta, Sara De Cima-Iglesias; Complejo Hospitalario Universitario A Coruña (A Coruña): Leticia Seoane- Quiroga, Alexandra Ceniceros-Barros; Hospital Universitario Miguel Servet (Zaragoza): Antonio L Ruiz-Aguilar, Luis M Claraco-Vega; Morales Meseguer University Hospital (Murcia): Juan Alfonso Soler, Maria del Carmen Lorente; Hospital Universitario del Henares (Coslada): Cecilia Hermosa, Federico Gordo; Complejo Asistencial De Palencia. Hospital Rio Carrión (Palencia): Miryam - Prieto-González, Juan B López-Messa; Fundación Jiménez Díaz (Madrid): Manuel P Perez, Cesar P Perez; Hospital Clínico Universitario Lozano Blesa (Zaragoza): Raquel Montoiro Allue; Hospital Verge de la Cinta (Tortosa): Ferran Roche- Campo, Marcos Ibañez-Santacruz; Hospital Universitario 12 De Octubre (Madrid): Susana - Temprano; Hospital Universitario Príncipe De Asturias (Alcalá De Henares, Madrid): Maria C Pintado, Raul De Pablo; Hospital Universitari Germans Trias I Pujol (Badalona): Pilar Ricart Aroa Gómez; Hospital Universitario Arnau De Vilanova De Lleida (Lleida): Silvia Rodriguez Ruiz, Silvia Iglesias Moles; Cst Terrassa (Barcelona): Ma Teresa Jurado, Alfons Arizmendi; Hospital Universitari Mútua Terrassa (Terrassa): Enrique A Piacentini; Hospital Universitario De Móstoles (Mostoles): Nieves Franco, Teresa Honrubia; Complejo Asistencial De Salamanca (Salamanca): Meisy Perez Cheng, Elena Perez Losada; Hospital General Universitario De Ciudad Real (Ciudad Real): Javier - Blanco, Luis J Yuste; Torrecardenas (Almeria): Cecilia Carbayo-Gorriz, Francisca G Cazorla-Barranquero; Hospital Universitario Donostia (San Sebastian): Javier G Alonso, Rosa S Alda; Hospital Universitario De Torrejón (Madrid): Ángela Algaba, Gonzalo Navarro; Hospital Universitario De La Princesa (Madrid): Enrique Cereijo, Esther Diaz-Rodriguez; Hospital Universitario Lucus Augusti (Lugo): Diego Pastor Marcos, Laura Alvarez Montero; Hospital Universitario Santa Lucia (Cartagena): Luis Herrera Para, Roberto Jimenez Sanchez; Hospital Universitario Severo Ochoa, Leganes (Madrid): Miguel Angel Blasco Navalpotro, Ricardo Diaz Abad; University Hospital Of Ntra. Sra. De Candelaria (Santa Cruz De Tenerife): Raquel Montiel Gonz á lez, D á cil Parrilla Toribio; Hospital Universitario Marques De Valdecilla (Santander): Alejandro G Castro, Maria Jose D Artiga; Hospital Infanta Cristina (Parla, Madrid): Oscar Penuelas ; Hospital General De Catalunya (Sant Cugat Del Valles): Tomas P Roser, Moreno F Olga; San Pedro De Alcántara (Cáceres): Elena
Gallego Curto, Rocío Manzano Sánchez; Sant Joan De Reus (Reus): Vallverdu P Imma, Garcia M Elisabet; Hospital Joan XXIII (Tarragona): Laura Claverias, Monica Magret; Hospital Universitario De Getafe (Madrid): Ana M Pellicer, Lucia L Rodriguez; Hospital Universitario Río Hortega (Valladolid): Jesús Sánchez-Ballesteros, Ángela González-Salamanca; Hospital Arquitecto Marcide (Ferrol,La Coruña): Antonio G Jimenez, Francisco P Huerta; Hospital General Universitario Gregorio Marañón (Madrid): Juan Carlos J Sotillo Diaz, Esther Bermejo Lopez;Hospital General De Segovia (Segovia): David D Llinares Moya, Alec A Tallet Alfonso; Hospital General Universitario Reina Sofia (Murcia): Palazon Sanchez Eugenio Luis, Palazon Sanchez Cesar; Complejo Hospitalario Universitario De Albacete (Albacete): Sánchez I Rafael, Corcoles G Virgilio; Hospital Infanta Elena (Valdemoro): Noelia N Recio;

SWEDEN: Sahlgrenska University Hospital (Gothenburg): Richard O Adamsson, Christian C Rylander; Karolinska University Hospital (Stockholm): Bernhard Holzgraefe, Lars M Broman; Akademiska Sjukhuset Uppsala (Uppsala): Joanna Wessbergh, Linnea Persson; Vrinnevisjukhuset (Norrköping): Fredrik Schiöler, Hans Kedelv; Linkoping University Hospital (Linköping): Anna Oscarsson Tibblin, Henrik Appelberg; Skellefteå Lasarett (Skellefteå): Lars Hedlund, Johan Helleberg; Karolinska University Hospital Solna (Stockholm): Karin E Eriksson, Rita Glietsch; Umeå University Hospital (Umeå): Niklas Larsson, Ingela Nygren; Danderyd Hospital (Stockholm): Silvia L Nunes, Anna-Karin Morin; Lund University Hospital (Lund): Thomas Kander, Anne Adolfsson;
SWITZERLAND: Chuv (Centre Hospitalier Universitaire Vaudois) (Lausanne): Lise Piquilloud; Hôpital neuchâtelois - La Chaux-De-Fonds (La Chaux-De-Fonds): Hervé O. Zender, Corinne Leemann-Refondini;
TUNISIA: Hopital Taher Sfar Mahdia (Mahdia): Souheil Elatrous; University Hospital Farhat Hached Sousse (Sousse): Slaheddine Bouchoucha, Imed Chouchene; CHU F.Bourguiba (Monastir): Islem Ouanes; Mongi Slim University Hospital, La Marsa (La Marsa): Asma Ben Souissi, Salma Kamoun;
TURKEY: Cerrahpasa Medical Faculty Emergency Intensive Care Unit (Istanbul): Oktay Demirkiran; Cerrahpasa Medical Faculty Sadi Sun Intensive Care Unit (Istanbul) : Mustafa Aker, Emre Erbabacan; Uludag University Medical Faculty (Bursa): Ilkay Ceylan, Nermin Kelebek Girgin; Ankara University Faculty of Medicine, Reanimation 3nd level ICU (Ankara): Menekse Ozcelik, Necmettin Ünal; Ankara University Faculty of Medicine, 2nd level ICU- postoperative ICU (Ankara): Basak Ceyda Meco; Istanbul Kartal Egitim Ve Arastirma Hastanesi (Istanbul): Onat O Akyol, Suleyman S Derman;
UNITED KINGDOM: Papworth Hospital (Cambridge): Barry Kennedy, Ken Parhar; Royal Glamorgan Hospital (Llantrisant): Latha Srinivasa; Royal Victoria Hospital-Belfast (Belfast): Lia McNamee, Danny McAuley; Jack Steinberg ICU of the King's College (London): Phil Hopkins, Clare Mellis; Frank Stansil ICU of the King's College Hospital (London): Vivek Kakar; ;Liver ICU of the King's College (London): Dan Hadfield; Christine Brown ICU of the King's College (London): Andre Vercueil; West Suffolk Hospital (Bury St Edmunds): Kaushik Bhowmick, Sally K Humphreys; Craigavon Area Hospital (Portadown): Andrew Ferguson, Raymond Mckee; Barts Health NHS Trust, Whipps Cross Hospital (Leytonstone): Ashok S Raj, Danielle A Fawkes; Kettering General Hospital, Foundation NHS Trust (Northamptonshire): Philip Watt, Linda Twohey; Barnet General Hospital (Barnet): Rajeev R JhaMatthew Thomas, Alex Morton, Varsha Kadaba; Rotherham General Hospital (Rotherham): Mark J Smith, Anil P Hormis; City Hospital, (Birmingham): Santhana G Kannan, Miriam Namih; Poole Hospital NHS Foundation Trust (Poole): Henrik Reschreiter, Julie Camsooksai; Weston General Hospital (Weston-Super-Mare): Alek Kumar, Szabolcs Rugonfalvi; Antrim Area Hospital (Antrim): Christopher Nutt, Orla Oneill; Aintree University Hospital (Liverpool): Colette Seasman, Ged Dempsey; Northern General Hospital (Sheffield): Christopher J Scott, Helen E Ellis; John Radcliffe Hospital (Oxford): Stuart Mckechnie, Paula J Hutton; St Georges Hospital (London): Nora N Di Tomasso, Michela N Vitale; Hillingdon Hospital (Uxbridge): Ruth 0 Griffin, Michael N Dean; The Royal Bournemouth & Christchurch NHS Foundation Trust (Bournemouth, Dorset): Julius H Cranshaw, Emma L Willett; Guys And St Thomas NHS Foundation Trust (London): Nicholas Ioannou, Gstt Severe Respiratory Failure Service ; Whittington Hospital (London): Sarah Gillis; Wexham Park Hospital (Slough): Peter Csabi; Western General Hospital (Edinburgh): Rosaleen Macfadyen, Heidi Dawson; Royal Preston Hospital (Preston): Pieter D Preez, Alexandra J Williams; Brighton And Sussex University Hospitals NHS Trust (Brighton): Owen Boyd, Laura Ortiz-Ruiz De Gordoa; East And North Herts NHS Trust (Stevenage): Jon Bramall, Sophie Symmonds; Barnsley Hospital (Barnsley): Simon K Chau, Tim Wenham; Prince Charles Hospital (Merthyr Tydfil): Tamas Szakmany, Piroska Toth-Tarsoly; University Hospital Of South Manchester NHS Foundation Trust (Manchester): Katie H Mccalman, Peter Alexander; Harrogate District Hospital (Harrogate): Lorraine Stephenson, Thomas Collyer; East And North Herts NHS Trust (Welwyn Garden City): Rhiannon Chapman, Raphael Cooper; Western Infirmary (Glasgow): Russell M Allan, Malcolm Sim; Dumfries And Galloway Royal Infirmary (Dumfries): David W Wrathall, Donald A Irvine; Charing Cross Hospital (London): Kim S Zantua, John C Adams; Worcestershire Royal Hospital (Worcester): Andrew J Burtenshaw, Gareth P Sellors; Royal Liverpool University Hospital (Liverpool): Ingeborg D Welters, Karen E Williams; Royal Alexandra Hospital (Glasgow): Robert J Hessell, Matthew G Oldroyd; Morriston Hospital (Swansea): Ceri E Battle, Suresh Pillai; Frimley Park Hospital (Frimley): Istvan - Kajtor, Mageswaran - Sivashanmugavel; Altnagelvin Hospital (Derry): Sinead C Okane, Adrian Donnelly; Buckinghamshire Healthcare NHS Trust (High Wycombe, Buckinghamshire): Aniko D Frigyik,
Jon P Careless; Milton Keynes Hospital (Milton Keynes): Martin M May, Richard Stewart; Ulster Hospital (Belfast): T John Trinder, Samantha J Hagan; University Hospital of Wales (Cardiff): Matt P Wise, Jade M Cole; Freeman Hospital (Newcastle Upon Tyne): Caroline C MacFie, Anna T Dowling;
URUGUAY: Hospital Español (Montevideo): Javier Hurtado, Nicolás Nin; Cudam (Montevideo): Javier

Hurtado; Sanatorio Mautone (Maldonado): Edgardo Nuñez; Sanatorio Americano (Montevideo): Gustavo Pittini, Ruben Rodriguez; Hospital De Clínicas (Montevideo): María C Imperio, Cristina Santos; Circulo Católico Obreros Uruguay- Sanatorio JPII (Montevido: Ana G. França, Alejandro EBEID; CASMU (Montevideo): Alberto Deicas, Carolina Serra

USA: Saint Louis University Hospital (St.Louis): Aditya Uppalapati, Ghassan Kamel; Beth Israel Deaconess Medical Center (Boston): Valerie M Banner-Goodspeed, Jeremy R Beitler; Memorial Medical Center (Springfield): Satyanarayana Reddy Mukkera, Shreedhar Kulkarni; Massachusetts General Hospital (Boston): Jarone Lee, Tomaz Mesar; University Of Cincinnati Medical Center (Cincinnati): John O Shinn Iii, Dina - Gomaa; Massachusetts General Hospital (Boston): Christopher Tainter, Jarone Lee; Massachusetts General Hospital (Boston): Tomaz Mesar, Jarone Lee; R Adams Cowley Shock Trauma Center (Baltimore): Dale J Yeatts, Jessica Warren; Intermountain Medical Center (Murray, Utah): Michael J Lanspa, Russel R Miller; Intermountain Medical Center (Murray, Utah): Colin K Grissom, Samuel M Brown; Mayo Clinic (Rochester): Philippe R Bauer; North Shore Medical Center (Salem): Ryan J Gosselin, Barrett T Kitch; Albany Medical Center (Albany): Jason E Cohen, Scott H Beegle; John H Stoger Hospital Of Cook County (Chicago, Il): Renaud M Gueret, Aiman Tulaimat; Albany Medical Center (Albany): Shazia Choudry ; University of Alabama at Birmingham (UAb) (Birmingham, AL): William Stigler, Hitesh Batra ; Duke University Hospital (Durham): Nidhi G Huff; Iowa Methodist Medical Center (Des Moines, Iowa): Keith D Lamb, Trevor W Oetting; Surgical & Neurosciences Intensive Care Unit of the University Of Iowa Hospitals And Clinics (Iowa City, Iowa): Nicholas M Mohr, Claine Judy; Medical Center of Louisiana at New Orleans (New Orleans, Louisiana): Shigeki Saito, Fayez M Kheir; Tulane University (New Orleans): Fayez Kheir; Critical Care Unit of the University Of Iowa Hospitals And Clinics (Iowa City, Iowa): Adam B Schlichting, Angela Delsing; University Of California, San Diego Medical Center (San Diego, Ca): Daniel R Crouch, Mary Elmasri; Uc San Diego Thornton Hospital (La Jolla): Daniel R Crouch, Dina Ismail; University Hospital (Cincinnati): Kyle R Dreyer, Thomas C Blakeman; University Hospital (Cincinnati): Kyle R Dreyer, Dina Gomaa; Tower 3B Medical ICU of Brigham and Women’s Hospital (Boston): Rebecca M Baron, Carolina Quintana Grijalba; Tower 8C Burn/Trauma ICU of Brigham and Women’s Hospital (Boston): Peter C Hou; Tower 8D Surgical ICU of Brigham and Women’s Hospital (Boston): Raghu Seethala; Tower 9C Neurosurgical ICU of Brigham and Women’s Hospital (Boston): Imo Aisiku; Tower 9D Neurological ICU of Brigham and Women’s Hospital (Boston): Galen Henderson; Tower 11C Thoracic ICU of Brigham and Women’s Hospital (Boston): Gyorgy Frendl; Shapiro 6W Cardiac Surgery ICU of Brigham and Women’s Hospital (Boston): Sen-Kuang Hou; Shapiro 9E Coronary Care Unit of Brigham and Women’s Hospital (Boston): Robert L Owens, Ashley Schomer;

SERBIA: Clinical Center of Serbia (Belgrade): Vesna Bumbasirevic, Bojan Jovanovic; Military Medical Academy (Belgrade): Maja Surbatovic, Milic Veljovic;

PRoVENT study

LIST OF PRoVENT NETWORK COLLABORATORS

AUSTRALIA: Canberra Hospital, Canberra: Frank Van Haren, Helen Rodgers; St Vincent’s Hospital Melbourne, Melbourne: Barry Dixon, Roger Smith; Concord Hospital, Sidney: Mark Kol, Helen Wong;

AUSTRIA: Vienna General Hospital, Vienna: Werner Schmid;

BELGIUM

UZ Leuven, Leuven: Greet Hermans, Helga Ceunen; AZ Sint-Jan Brugge-Oostende AV, Brugge: Marc Bourgeois, Nathalie Anquez; Ghent University Hospital, Gent: Johan Decruyenaere, Luc DeCrop;

BRAZIL; Hospital Israelita Albert Einstein, São Paulo: Ary Serpa Neto, Rafaella Souza dos Santos

Hospital Renascentista, Pouso Alegre: Daniel Beraldo; Hospital Montenegro, Montenegro: Moreno Calcagnotto dos Santos, Jose Augusto Santos Pellegrini; Hospital Vitória Apart, Vitória: Claudio Piras; Hospital Nossa Senhora da Conceição, Porto Alegre: Vanessa Oliveira; Hospital Moinhos de Ventos, Porto Alegre: Carlos Munhoz, Ana Carolina Peçanha ; Hospital Vivalle, São José dos Campos: Fernando José da Silva Ramos; Hospital Nereu Ramos, Florianópolis: Israel Maia, Marina Bahl; Hospital Alvorada Taguatinga, Taguatinga: Rodrigo Biondi, Daniel Prado; Universidade Federal de Mato Grosso do Sul, Campo Grande: Sérgio Felix Pinto, Jean Salgado

Universidade Federal de São Paulo – Escola Paulista de Medicina, São Paulo: Luis Fernando Falcão, Tiago Macruz; Hospital do Coração, São Paulo: Alexandre Biasi Cavalcanti, Marcelo Luz Pereira Romano, Kessia Ruas; Hospital Universitário São Francisco, Bragança Paulista: Giovana Colozza Mecatti; Hospital UNIMED Vitória, Vitória: Eliane Bernadete Caser, Isabela Ambrósio Gava

CHILE: Hospital Santiago Oriente – Dr Luis Tisné Brousse, Santiago: Nicolás Carreño; Hospital Clinico Magallanes, Punta Arenas: Mauricio Morales, Rossana Avendaño; Hospital Dr Gustavo Fricke, Viña Del Mar: Stefania Aguirre

CROATIA: Clinical Hospital Dubrava, Zagreb: Andrej Sribar, Vlasta Klaric; University of Osijek, Osijek: Sonja Skilijic; University Hospital Merkur, Zagreb: Matea Bogdanovic Dvorscak, Marijana Krkusek; 'Dr Josip Bencevic' General Hospital, Slavonski Brod: Matija Jurjevic; Split University Hospital Center, Split: Nenad Karanovic; General Hospital Zadar, Zadar: Tatjana Simurina
CZECH REPUBLIC: University Hospital Brno – Medical Faculty of Msaryk University, Brno: Petr Stourac, Milan Kratochvil; University Hospital Ostrava, Ostrava: Jan Máca
GERMANY; University Hospital Leipzig, Leipzig: Hermann Wrigge, Christian Schlegel; University Hospital Dusseldorf, Dusseldorf: Tanja A Treschan, Maximilian Schaefer, Akut Aytulun and Peter Kienbaum
IRELAND: Galway University Hospital, Galway: Kevin Clarkson, Rola Jaafar; St James's Hospital, Dublin: Daniel Collins; Cork University Hospital, Cork: Robert Plant
ITALY: IRCCS 'Casa Sollievo Della Sofferenza, San Giovanni Rotondo: Giuseppe Melchionda, Eduardo Di Lauro; Policlinico P Giaccone – University of Palermo, Palermo: Andrea Cortegiani, Vincenzo Russotto; Vito Fazzi Hospital, Lecce: Raffaele Caione, Donatella Mestria; Università Degli Studi di Ferrara, Ferrara: Carlo Alberto Volta, Savino Spadaro; Spedali Civili di Brescia – University of Brescia, Brescia: Marco Botteri, Elisa Seghelini; Sassari University Hospital, Sassari: Luca Brazzi, Gabriele Sales; Ospedali Riuniti – University of Foggia, Foggia: Davide D'Antini, Gilda Cinnella, Lucia Mirabella; IRCCS San Martino – University of Genoa, Genoa: Paolo Pelosi, Alexandre Molin; Insubria University of Varese, Varese: Paolo Severgnini, Alessandro Bacuzzi, Lorenzo Peluso; ASL Bari – Monopoli Hospital, Monopoli: Pasquale Verrastro, Pasquale Raimondo
KOSOVO: University Clinical Center of Kosovo, Prishtina: Agreta Gecaj-Gashi
NETHERLANDS: University of Amsterdam – Academic Medical Center, Amsterdam: Marcus J Schultz, Fabienne D Simonis; VU University Medical Center, Amsterdam: Pieter Roel Tuinman, Erna Alberts, Ingrid van den Hul; Leiden University Medical Center, Leiden: Robert BP de Wilde
Medisch Centrum Leeuwarden, Leeuwarden: Michael Kuiper, Matty Koopmans
TURKEY: Tepecik Training and Research Hospital, Izmir: Isil Kose, Çiler Zincircioglu; Ataturk University, Erzurum: Nazim Dogan; Celal Bayar University, Manisa: Demet Aydin; Ozel Primer Hospital, Gaziantep: Ahmet Sukru Denker; Kirikkale University, Kirikkale: Unase Buyukkocak; Fatih Sultan Mehmet Egitim ve Arastirma Hastanesi, Instabul: Nur Akgun, Güldem Turan; Instabul Medicine Faculty, Instanbul: Evren Senturk, Zerrin Demirtürk, Perihan Ergin Özcan; Haydarpasa Numune Egitim ve Arastirma Hastanesi, Instanbul: Osman Ekinci; Kanuni Education and Training Hospital, Instanbul: Sedat Saylan; Bakirkoy Dr Sadi Konuk Egitim ve Arastirma Hastanesi, Bakirkoy: Gulay Eren; Ondokuz Mayis University, Samsun: Fatma Ulger, Ahmet Dilek; Karadeniz Teknik University, Trabzon: Hulya Ulusoy; Yüzüncü Yil University, Van: Ugur Goktas, Lokman Soyoral; Çanakkale Onsekiz Mart University, Çanakkale: Huseyin Toman; Mardin Devlet Hastanesi, Mardin Merkez: Yavuz Orak; Uludag University Faculty of Medicine, Bursa: Feda Kahveci
UNITED KINGDOM: Sheffield Teaching Hospital, Sheffield: Gary H Mills, Angela Pinder, Rachel Walker, Jonathan Harrison; Aintree University Hospital NHS Foundation Trust, Liverpool: Jane Snell, Colette Seasman; Central Manchester University Hospital, Manchester: Rachel Pearson, Michael Sharman; Gloucestershire Hospitals NHS Trust, Gloucester: Claire Kaloo, Natalie Bynorth, Kelly Matthews, Chloe Hughes; The Mid Yorkshire Hospitals NHS Trust, Wakefield: Alastair Rose, Karen Simeson; Milton Keynes Hospital NHS Foundation Trust, Milton Keynes: Lotta Niska, Nathan Huneke, Jane Adderly, Cheryl Padilla-Harris, Rebecca Oliver; North Tees and Hartlepool NHS Foundation Trust , Hartlepool: Farooq Brohi, Natalie Wilson, Helen Talbot, Deborah Wilson, Deborah Smith; Salford Royal NHS Foundation Trust, Salford: Paulo Dark, Tracey Evans, Nicola Fisher; South Devon Healthcare NHS Foundation Trust, Torquay: Jane Montgomery, Pauline Fitzell; South Tees Hospital NHS Foundation Trust, Middlesbrough: Christoph Muench, Keith Hugill, Emanuel Cirstea; University Hospitals of South Manchester NHS Foundation Trust, Manchester: Andrew Bentley, Katie Lynch; Ashford and St Peters Hospital NHS Foundation Trust, Chertsey: Ian White, Jonathan Cooper, Melinda Brazier, Michael Devile, Michael Parris, Pardeep Gill, Tasmin Patel; Basingstoke and North Hampshire NHS Foundation, Basingstoke: John Criswell, Dawn Trodd Denise Griffin, Jane Martin, Caroline Wreybrown; Bristol Royal Infirmary, Bristol: Jeremy Bewley, Katie Sweet, Lisa Grimmer, Marta Kozlowski, Shanaz James; County Durham and Darlington NHS Foundation Trust, Darlington: James Limb, Amanda Cowton; Derby Hospitals NHS Foundation Trust, Derby: David Rogerson, Charlotte Downes, Susan Melbourne, Ryan Humphries; Dorset County Hospital, Dorchester: Mark Pulletz, Sarah Moreton, Stephanie Janes; East Sussex Healthcare Trust, East Sussex: Andrew Corner; Gateshead Health NHS Foundation Trust, Gateshead: Vanessa Linnett, Jenny Ritzema; Great Western Hospital, Swindon: Malcolm Watters, Steve Windebank, Shailaja Chenna; Ipswich Hospital NHS Trust, Ipswich: Richard Howard-Griffin, Kate Turner, Sheeba Suresh, Heather Blaylock, Stephanie Bell; James Paget University Hospital NHS Foundation Trust, Great Yarmouth: Karl Blenk,

Lynn Everett; Kings College Hospital, London: Phil Hopkins, Clare Mellis, Daniel Hadfield, Clair Harris, Alexandre Chan, Sian Birch; Medway NHS Foundation Trust, Gillingham: Claire Pegg, Catherine Plowright, Lucy Cooper, Tom Hatton; The Newcastle Upon Tyne Hospitals NHS Foundation Trust, Newcastle Upon Tyne: Iain McCullagh, Stephen Wright, Carmen Scott, Christine Boyd; North Cumbria University Hospitals NHS Trust, Hensingham: Mark Holliday, Una Poultney, Hannah Crowther, Sarah Thornthwaite; North Devon Healthcare NHS Trust, Barnstaple: Nigel Hollister, Jane Hunt, Amanda Skinner; University Hospital of North Staffordshire NHS Trust, Stoke on Trent: Ramprasad Matsa, Ruth Salt, Claire Matthews; Poole Hospital NHS Foundation Trust, Poole: Henrik Reschreiter, Julie Camsooksai, Nicola Venner, Helena Barcraft-Barnes, Lee Tbaily; Portsmouth Hospital NHS Trust, Portsmouth: David Pogson, Johanna Mouland, Steve Rose, Nicola Lamb, Nicholas Tarmey, John Knighton; Queen Victoria Hospital NHS Foundation Trust, East Grinstead: Julian Giles, Debbie Weller, Isabelle Reed; The Rotherham NHS Foundation Trust, Rotherham: Anil Hormis, Sallyane Pearson, Meredith Harris, Joanne Howe, Anil Hormis; Royal Cornwall Hospital, Truro: Jonathan Paddle, Karen Burt; Royal Liverpool and Broadgreen University Hospitals NHS Trust, Liverpool: Ingeborg Welters, Anna Walker, Laura Youds, Sam Hendry, David Shaw, Karen Williams; Royal Shrewsbury Hospitals NHS Trust, Shrewsbury: Robin Hollands, Mandy Carnahan, Johanna Stickley, Claire Miller, Denise Donaldson, Louise Tonks; Royal Surrey County Hospital NHS Foundation Trust, Guildford: Ben Creagh-Brown, Daniel Hull; Royal Sussex County Hospital, Brighton: Owen Boyd, Laura Ortiz-Ruiz
The Royal Wolverhampton NHS Trust, Wolverhampton: Shammer Gopal, Stella Metherell, Hazel Spencer; South Tyneside NHS Foundation Trust, South Sheilds: Christian Frey, Carly Brown, Gayle Clifford; St Georges Hospital London, London: Susannah Leaver, Christine Ryan, Johannes Mellinghoff, Sarah Prudden, Helen Green; City Hospitals Sunderland NHS Foundation Trust, Sunderland: Alistair Roy, Julie Furneval, Adam Bell; The Walton Centre NHS Foundation Trust, Liverpool: Sandeep Lakhani, Lousie Fasting, Lorna Murray; Cambridge University Hospitals NHS Foundation (Addenbrookes), Cambridge: Kobus Preller, Amy McInerney; Chesterfield Royal Hospital NHS Foundation Trust, Chesterfield: Sarah Beavis, Amanda Whileman, Julie Toms, Sue Glenn; Colchester Hospital University NHS Foundation Trust, Colchester: Mohamed Ramali, Alison Ghosh, Clare Bullock, Lisa Barrell; Countess of Chester Hospital NHS Foundation Trust, Chester: Eoin Young, Helen Robertson, Maria Faulkner; Plymouth Hospitals NHS Trust, Plymouth: Peter MacNaughton, Susan Tyson; Sherwood Forest Hospitals NHS Foundation Trust, Sutton-in-Ashfield: Paul Pulak, Terri-Ann Sewell; Wirral University Teaching Hospital NHS Foundation Trust, Wirral: Christopher Smalley, Reni Jacob

URUGUAY: Hospital de Clinicas, Montevideo: Cristina Santos, Pedro Alzugaray

UNITED STATES OF AMERICA: Massachusetts General Hospital, Boston: Marcos F Vidal Melo, Kristen Joyce, Joseph Needleman

PRoVENT–iMiC study

PRoVENT-iMiC SITE INVESTIGATORS BY COUNTRY (in alphabetical order)

BANGLADESH: AKM Shamsul Alam, Syeda Nafisa Khatoon, Mohammed Abdur Rahman Chowdhury (Chittagong Medical College Hospital, Chittagong, Bangladesh); Debabrata Banik, Montosh Kumar Mondol, Sakibur Rahman Bhuiyan (Bangabandhu Sheikh Mujib Medical University, Dhaka, Bangladesh); Areef Ahsan, Suraiya Nazneen, Rozina Sultana, Tarikul Hamid (BIRDEM General Hospital, Dhaka, Bangladesh); Mozaffer Hossain, Syed Tariq Reza, Muhammad Asaduzzaman, Mohammad Salim, (Dhaka Medical College Hospital, Dhaka, Bangladesh); Abu Hena Mostafa Kamal, Sheikh Mohammed Taher, Taohidul Majid Taohid, Pranab Karmaker (Rajshahi Medical College Hospital, Rajshahi, Bangladesh); Sabyasachi Roy, Shantanu Das, Sohel Ahmesd Sarkar, Monju Lal Dutta, Poulomi Roy (Sylhet MAG Osmani Medical College Hospital, Sylhet, Bangladesh)

INDIA: Bhuvana Krishna, Sriram Sampath (St. John's Medical College, Bangalore); Chinni Krishna Kasi, Rajyabardhan Pattnaik, (Ispat General Hospital, Rourkela, India); Shiva Iyer, Jignesh Shah (Bharati Vidyapeeth Medical College, Pune, India); Anand Dongre (Swastik Critical Care, Nagpur, India)

IRAN: Navid Nooraei (Modarres Hospital, Tehran, Iran); Reza Hashemian, Reza Raessi Estabragh, Majid Malekmohammad (Masih Daneshvari Hospital, Tehran, Iran); Batoul Khoundabi (Red Crescent Society of the Islamic Republic of Iran, Tehran, Iran); Maziar Mobasher (Tehran Pars Hospital, Tehran, Iran)

MALAYSIA: Nor'azim Mohd Yunos, Mahazir Kassim, Voon Chern Min, Stanis Sutharsa Das, Siti Nur Suhaila Azauddin, Dharshinie Dorasamy, (Hospital Sultanah Aminah Johor Bahru, Malaysia); Tai Li Ling (Hospital Kuala Lumpur, Kuala Lumpur, Malaysia); Mohd Basri Mat Nor, Nurhafizah Zarudin (International Islamic University Medical Centre, Kuantan, Malaysia); Mohd Shahnaz Hassan,

Mohamad Fadhil Hadi Jamaluddin, Mohamad Irfan Bin Othman Jailani, (University of Malaya Medical Centre, Kuala Lumpur, Malaysia)
MALDIVES: Hassan Moosa, Hisham Ahmed Imad (Indira Gandhi Memorial Hospital, Malé, Maldives)
NEPAL: Gyan Kayastha, Aaradhana Adhikari, Raju Pangeni (Patan Academy of Health Sciences, Kathmandu, Nepal)
PAKISTAN: Sonia Joseph (Allied Hospital, Faisalabad, Pakistan); Aftab Akhtar, Aayesha Qadeer (Shifa International Hospital, Islamabad, Pakistan); Iqbal Memon, Syed Muneeb Ali (Pakistan Institute of Medical Sciences, Islamabad, Pakistan); Farah Idrees, Saima Kamal (Aga Khan University, Karachi, Pakistan); Sadaf Hanif, Atta Ur Rehman (Patel Hospital, Karachi, Pakistan); Arshad Taqi, Tanveer Hussain (National Hospital and Medical Center, Lahore, Pakistan); Ahmed Farooq (Doctor's Hospital, Lahore, Pakistan); Saleh Khaskheli (Peoples Medical College Hospital, Nawabshah, Pakistan); Muhammad Hayat (North West General Hospital, Peshawar, Pakistan)
SRI LANKA: Upeka Samaranayake (Anuradhapura Teaching Hospital, Anuradhapura, Sri Lanka); S. Mathanalagan (Base Hospital, Batticaloa, Sri Lanka); Asoka Gunaratne (Colombo South Teaching Hospital, Colombo, Sri Lanka); Kanishka Indraratna, Nimangee Mithraratne, Kaushila Thilakasiri, Chamila Pilimatalawwe, Y. A. Hasitha Dilhani (General Hospital Sri Jayawardenapura, Colombo, Sri Lanka); Marie Fernando, Kumudini Ranatunge (National Hospital Sri Lanka – SICU, Colombo, Sri Lanka); Loranthi Samarasinghe, Manori Vaas (Lanka Hospital, Colombo, Sri Lanka); Manoj Edirisooriya (National Hospital Sri Lanka – MICU, Colombo, Sri Lanka); Chaturani Sigera (Network for improving Critical Care Systems and Training, Colombo, Sri Lanka); Janaki Arumoli (Jaffna Teaching Hospital, Jaffna, Sri Lanka); Kesharie De Silva (Karapitiya Teaching Hospital, Galle, Sri Lanka); Bimal Kudavidanage (Base Hospital, Kegalle, Sri Lanka); Visanthi Pinto (Peradenyia University Hospital, Peradenyia, Sri Lanka); Lakshman Dissanayake (Puttalam Base Hospital, Puttalam, Sri Lanka) –
THAILAND: Napplika Kongpolprom (King Chulalongkorn Memorial Hospital, Chulalongkorn University, Bankgok, Thailand); Hisham Ahmed, Udomsak Silachamroon (Hospital for Tropical Diseases, Mahidol University, Bangkok, Thailand); Prapaporn Pornsuriyasak, Tananchai Petnak, Pongsasit Singhatas, Viratch Tangsujaritvijit (Ramathibodi Hospital, Bangkok, Thailand); Suthat Rungruanghiranya (Srinakharinwirot University, Ongkarak, Thailand); Annop Piriyapatsom (Sriraj Hospital, Bankok, Thailand); Kaweesak Chittawatanarat, Kanokkarn Juntaping (SICU, Department of Surgery, Faculty of Medicine, Chiang Mai University, Maharaj Nakorn Chiang Mai Hospital, Chiang Mai, Thailand); Konlawij Trongtrakul, Poungrat Thungtitikul (Vajira Hospital, Bangkok, Thailand); Pattraporn Tajarernmuang (Chiang Mai Hospital – MICU, Chiang Mai, Thailand); Sunisa Chatmongkolchart, Rungsun Bhurayanontachai, Osaree Akaraborworn, Asma Navasakulpong (Prince of Songkla University, Hatyai, Thailand); Karjbundid Surasit (Nakornping Hospital, Chiang Mai, Thailand);
VIET NAM: Behzad Nadjm, Vu Quoc Dat, Nguyen Thi Thanh Ha, Nguyen Van Kinh (National Hospital for Tropical Diseases, Hanoi, Viet Nam); Duong Bich Thuy (Hospital for Tropical Diseases, Ho Chi Minh City, Vietnam); Lam Minh Yen, Louise Thwaites (Oxford University Clinical Research Unit, Ho Chi Minh City, Viet Nam).

MEMBERS OF THE PRoVENT-iMiC STEERING COMMITTEE

Luigi Pisani (Mahidol-Oxford Tropical Medicine Research Unit, Bangkok, Thailand; Amsterdam University Medical Centers, location AMC, Amsterdam, The Netherlands); Ary Serpa Neto (Amsterdam University Medical Centers, location AMC, Amsterdam, The Netherlands; Hospital Israelita Albert Einstein, São Paulo, Brazil); Anna Geke Algera (Amsterdam University Medical Centers, location AMC, Amsterdam, The Netherlands); Salvatore Grasso (Bari University Policlinic Hospital, University of Bari, Bari, Italy); Frederique Paulus (Amsterdam University Medical Centers, location AMC, Amsterdam, The Netherlands); Marcelo Gama de Abreu (University Hospital Carl Gustav Carus, and Technical University Dresden, Dresden, Germany); Paolo Pelosi (San Martino Policlinico Hospital - IRCCS for Oncology, University of Genoa, Genoa, Italy); Arjen M. Dondorp (Mahidol University, Bangkok, Thailand); Marcus J. Schultz (Mahidol-Oxford Tropical Medicine Research Unit, Bangkok, Thailand; Amsterdam University Medical Centers, location AMC, Amsterdam, The Netherlands; University of Oxford, Oxford, UK).

MEMBERS OF THE PRoVENT-iMiC WRITING COMMITTEE

Luigi Pisani (Mahidol–Oxford Tropical Medicine Research Unit, Bangkok, Thailand; Amsterdam University Medical Centers, location AMC, Amsterdam, The Netherlands); Ary Serpa Neto (Hospital Israelita Albert Einstein, São Paulo, Brazil; and Faculdade de Medicina do ABC, Santo André, Brazil); Arjen M. Dondorp (Mahidol–Oxford Tropical Medicine Research Unit, Bangkok, Thailand); Marcus J. Schultz (Mahidol–Oxford Tropical Medicine Research Unit, Bangkok, Thailand; Amsterdam University Medical Centers, location AMC, Amsterdam, The Netherlands, University of Oxford, Oxford, UK).

FUNDING OF INDIVIDUAL STUDIES

ERICC was funded by the Research and Education Institute from Hospital Sírio-Libanês, São Paulo, the D'Or Institute for Research and Education, Rio de Janeiro, and supported by the Brazilian Research in Intensive Care Network (BRICNet), Brazil. LUNG SAFE was funded by the European Society of Intensive Care Medicine (ESICM) by St Michael's hospital, Toronto, Canada, and by the University of Milan-Bicocca, Milan, Italy; PRoVENT was funded by the Amsterdam University Medical Centers, location 'AMC'; PRoVENT–iMiC was funded by the Mahidol–Oxford Tropical Medicine Research Unit (MORU), Bangkok, Thailand, and the Amsterdam University Medical Centers, location 'AMC'.
